# Probing the core metabolism of *Cereibacter sphaeroides* by transposon mutagenesis

**DOI:** 10.1128/jb.00306-25

**Published:** 2025-10-30

**Authors:** Birgit E. Alber, Jessica A. Adair, Marie Asao, Suzy Bangudi, Samuel N. Kotran, Kathleen Sandman

**Affiliations:** 1 Department of Microbiology, Ohio State University 215854, Columbus, Ohio, USA; Geisel School of Medicine at Dartmouth, Hanover, New Hampshire, USA

**Keywords:** central carbon metabolism, 3-hydroxypropionate metabolism, pyruvate carboxylase, pyruvate dehydrogenase, secondary L-glutamate TAXI transport system

## Abstract

During phototrophic growth, *Cereibacter sphaeroides* can use several carbon substrates that are central carbon intermediates (e.g., succinate and L-malate) or that require only a few steps to enter central carbon metabolism (e.g., acetate and D-malate). In addition, with light as the energy source, the carbon substrate provided will function as a carbon source for cell carbon synthesis only. Therefore, *C. sphaeroides* is ideally suited to understand the changes necessary to switch between different carbon sources and, consequently, to redirect carbon flow in central carbon metabolism. This study describes *C. sphaeroides* transposon mutants that have lost the ability to use one or more of the organic carbon sources 3-hydroxypropionate, acetate, L-malate, propionate/HCO_3_^–^, butyrate/HCO_3_, L-lactate, D-lactate, D-malate, and L-glutamate. Pyruvate carboxylase and pyruvate dehydrogenase were confirmed to connect the precursor metabolite pools of pyruvate and oxaloacetate or acetyl-CoA, respectively, as was the ethylmalonyl-CoA pathway connecting acetyl-CoA and oxaloacetate pools. Transposon and in-frame deletion mutants suggest that 3-hydroxypropionate is oxidized to CO_2_ and acetyl-CoA, involving a malonate semialdehyde dehydrogenase. The presence of this oxidative route makes pyruvate dehydrogenase dispensable during 3-hydroxypropionatedependent growth. Therefore, acetyl-CoA represents a second entry point into central carbon metabolism for 3-hydroxypropionate besides succinyl-CoA, and it is proposed that the simultaneous functioning of the two routes minimizes transiently produced CO_2_/HCO_3_^–^. Another significant outcome of this study is the identification of genes encoding a L-glutamate TRipartite ATP-independent transporter, which was characterized biochemically 30 years ago.

**IMPORTANCE:**

Several aspects of the process of carbon assimilation, defined as the conversion of a carbon source into cell carbon, are conserved throughout life. For example, common building blocks give rise to proteins and nucleic acids, and the carbon for building blocks, cofactors, and secondary metabolites is derived from common precursor metabolites such as acetyl-CoA, pyruvate, or oxaloacetate. Using carbon substrates that require only one or a few steps to enter central carbon metabolism facilitates insights into the changes that occur to accommodate growth with different carbon substrates. In this study, transposon mutants that affect carbon flow in the core metabolism of *Cereibacter sphaeroides* were identified. Apparent redundancies of pathways can be explained by the need to maintain overall redox balance.

## INTRODUCTION

*Cereibacter sphaeroides* (formerly *Rhodopseudomonas spheroides* and *Rhodobacter sphaeroides* [1]) strain 2.4.1 was isolated over 80 years ago and has since been used as a model organism to study energy conservation by cyclic photophosphorylation and by oxidative phosphorylation, because growth of the purple non-sulfur bacterium is possible both anaerobically in the light and aerobically in the dark (2). It was noted early on that purple bacteria, in general, grow not only photoautotrophically (photosynthetically) but can also assimilate a variety of organic carbon substrates in the light (3). During this so-called photoheterotrophic growth mode, dependent on the oxidation state of the carbon source, CO_2_/HCO_3_^–^ is either a required co-substrate (e.g., for propionate), or CO_2_/HCO_3_^–^ is a necessary product (*e.g*., for malate), but typically no organic acids are formed as end-products of their metabolism, and therefore, purple bacteria display an economically streamlined use of their carbon sources (4).

**Fig 1:**
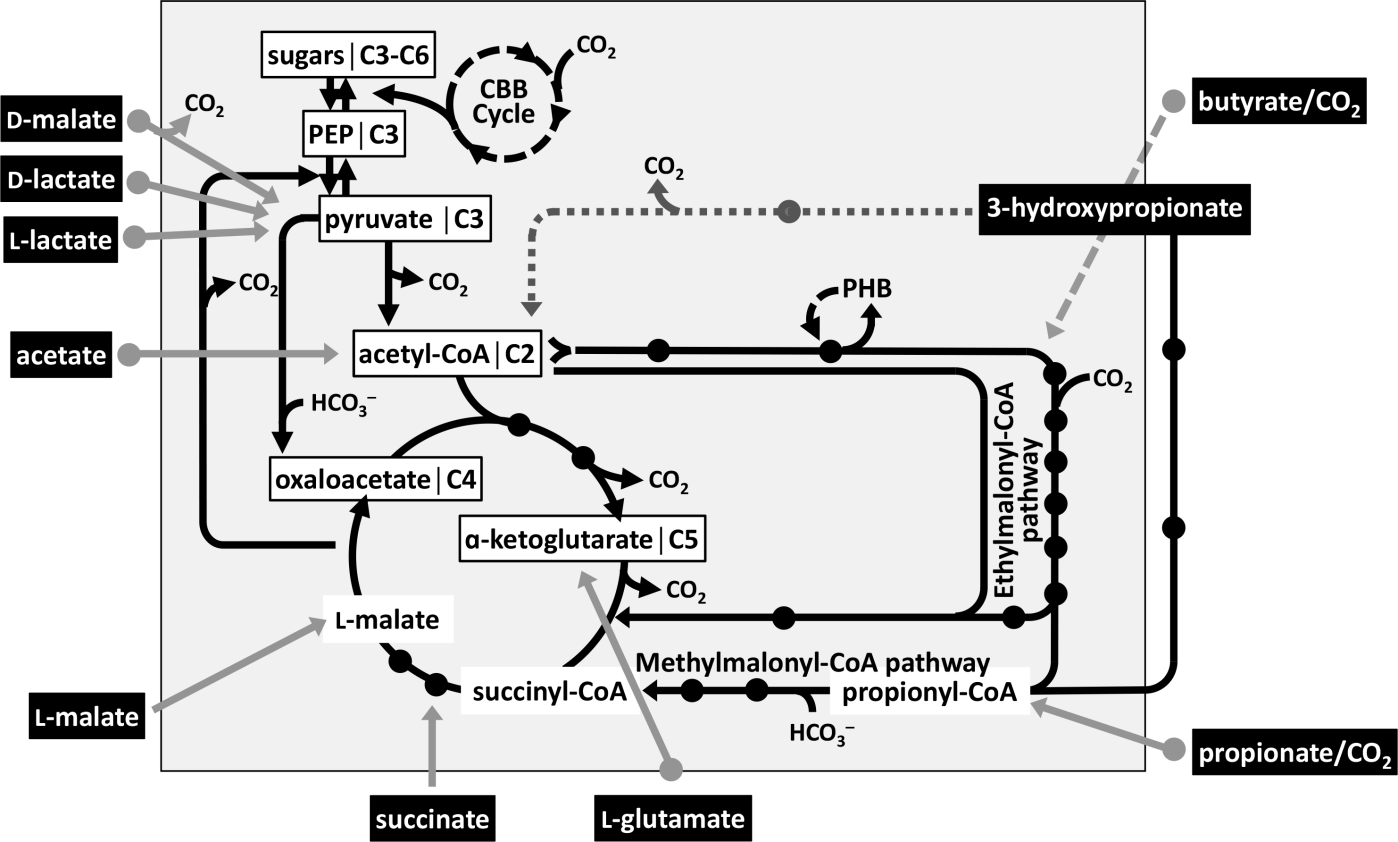
The core metabolism of *C. sphaeroides*. Entry points into central carbon metabolism for different carbon substrates (filled boxes) are indicated, based on genome annotation or experimental results. Precursor metabolites are highlighted in white boxes and are central carbon intermediates that provide carbon for biosynthesis pathways. Filled black dots indicate known central carbon intermediates. Oxidants and reductants are not considered; however, the requirement at each step can be inferred by the change in the total oxidation state of the carbon. The broken arrow for the conversion of butyrate indicates that the entry point into central carbon metabolism is not known. Genetic evidence for a so-called oxidative route (dotted line) for the conversion of 3-hydroxypropionate to acetyl-CoA and CO_2_ via malonate semialdehyde is presented in this study. A so-called reductive route from 3-hydroxypropionate and bicarbonate to succinyl-CoA has been described previously (5, 6). For *C. sphaeroides*, there are two possibilities to connect C4- to C3-pools: either from oxaloacetate to phosphoenolpyruvate (via PEP carboxykinase) or from L-malate to pyruvate (via malic enzyme). PHB, polyhydroxybutyrate; CBB cycle, Calvin Benson-Bassham cycle.

To further our understanding of the core metabolism, of particular interest is the ability of *C. sphaeroides* to use several carbon substrates that only require a few steps to be converted to central carbon intermediates. These carbon substrates include acetate, propionate/HCO_3_^–^, butyrate/HCO_3_^–^, L-lactate, D-lactate, D-malate, L-glutamate, and 3-hydroxypropionate (Fig. 1). L-Malate and succinate are also used as carbon substrates by *C. sphaeroides* and are intermediates of the central carbon metabolism. During aerobic respiratory growth, the carbon substrate will be used both as a carbon source, i.e., the carbon is converted to cell carbon in assimilation, and as a source of energy, i.e., part of the carbon substrate is oxidized to CO_2_/HCO_3_^–^ with the electrons transferred to oxygen for energy conservation. During anaerobic photoheterotrophic growth, however, all the carbon of a given carbon substrate is available for assimilation, as light is the source of energy. In the absence of an additional electron donor or electron acceptor, one also needs to consider the fate of electrons for the conversion of the carbon source to cell carbon. If the carbon in the carbon substrate is more oxidized than that of the cell carbon, part of the carbon substrate will be oxidized to CO_2_ to provide the additional electrons needed. However, if the average carbon of the carbon substrate is more reduced than the average cell carbon, CO_2_/HCO_3_^–^ is a required co-substrate (4). Consequently, the relative amount of CO_2_/HCO_3_^–^ released or co-assimilated depends on the oxidation state of the cell carbon to ensure redox balance, meaning that overall, all electrons are accounted for. Because several carboxylation and decarboxylation reactions occur in central carbon metabolism (Fig. 1), carbon flow in central carbon metabolism needs to be directed to ensure overall carbon- and redox-balance and to allow for the net fixation or release of the appropriate amount of CO_2_/HCO_3_^–^ for the different carbon substrates.

The metabolism of several carbon substrates has been established for *C. sphaeroides*. Acetate is activated to acetyl-CoA, and assimilation of acetyl-CoA proceeds via the ethylmalonyl-CoA pathway, a reaction sequence that converts three acetyl-CoA, one CO_2_, and one bicarbonate molecule to L-malate and succinyl-CoA (7). Propionate is activated to propionyl-CoA, and assimilation proceeds via the methylmalonyl-CoA pathway, a reaction sequence that converts propionyl-CoA and HCO_3_^–^ to succinyl-CoA (8). Propionyl-CoA is also an intermediate in acetyl-CoA assimilation, and the methylmalonyl-CoA pathway, therefore, is part of the ethylmalonyl-CoA pathway (Fig. 1). A transcriptional regulator PccR, of the short-chain fatty acyl coenzyme A regulator (ScfR) family, was identified and was shown to control the expression of genes encoding propionyl-CoA carboxylase, a key enzyme of the methylmalonyl-CoA pathway (9). L-Lactate and D-lactate are expected to be oxidized to pyruvate as the entry point into central carbon metabolism. A membrane-bound, NAD(P)-independent L-lactate dehydrogenase from *C. sphaeroides* has been biochemically characterized; however, the gene(s) encoding the enzyme has not been determined (10). The carbon substrate D-malate is predicted to be oxidized to CO_2_ and pyruvate by an NAD^+^-dependent decarboxylating malate dehydrogenase. The metabolism of butyrate has not been studied extensively, but assimilation of butyryl-CoA likely proceeds via the ethylmalonyl-CoA pathway. More recently, the assimilation of 3-hydroxypropionate by *C. sphaeroides* has been investigated, and a so-called reductive route has been established based on genetic and biochemical data, where 3-hydroxypropionate is converted to propionyl-CoA, which is further assimilated via the methylmalonyl-CoA pathway (5, 6). Surprisingly, during photoheterotrophic growth with 3-hydroxypropionate, carbon flux through the ethylmalonyl-CoA pathway occurs, and it was proposed that the ethylmalonyl-CoA pathway allows for the appropriate amount of CO_2_/HCO_3_^–^ being released from the cell to ensure a redox balance, meaning that electrons provided by the carbon substrate are recoverable in the cell mass (11).

Here, *C. sphaeroides* transposon mutants that had lost the ability to use one or more of the organic carbon sources acetate, propionate/HCO_3_^–^, butyrate/HCO_3_^–^, L-lactate, D-lactate, D-malate, L-glutamate, and 3-hydroxypropionate were isolated. We present genetic evidence that the metabolism of 3-hydroxypropionate involves a pathway, where 3-hydroxypropionate is oxidized to CO_2_ and acetyl-CoA. This oxidative route represents an entry point into central carbon metabolism, in addition to the reductive route, where 3-hydroxypropionate and HCO_3_^–^ are converted to succinyl-CoA. Another outcome of the forward genetic screen for *C. sphaeroides* is the confirmation of pyruvate carboxylase as the enzyme that connects three-carbon to four-carbon central carbon intermediates. For acetate as a carbon source, surprisingly, growth with acetate was not abolished when the methylmalonyl-CoA pathway, as part of the ethylmalonyl-CoA pathway, was interrupted by mutations in genes encoding propionyl-CoA carboxylation. Finally, the genes encoding an L-glutamate TRipartite ATP-independent transport (TRAP) system, which was previously biochemically characterized (12), could be inferred based on growth phenotypes.

## RESULTS

To study the genetic requirements for the use of different carbon substrates, random transposon mutant libraries of *C. sphaeroides* were screened, and mutants that had lost the ability to use a specific carbon substrate were isolated. A suicide plasmid, developed by Larsen et al. (13), containing a mini-transposon and encoding a highly active transposase, was introduced into *C. sphaeroides*. A total of more than 150 libraries with over 200,000 mutants were screened, and the transposon insertion sites for 399 mutants affecting 266 different genes were identified. A primary screen was conducted using plates that were incubated aerobically in the dark, and a secondary screen included anaerobic photoheterotrophic conditions, too. This report includes only those genes with at least two independent transposon insertions within the same genomic locus and whose strains show a consistent and reproducible phenotype. Compromised growth is defined as significantly less growth compared to the growth of other mutants on the same plate by visual inspection. A negative growth phenotype was determined if growth on the control plate was positive, but no colonies (or a few individual colonies on the diagonal streak as a sign of possible suppression) on one or more of the other plates were detected (for further details, please see the Materials and Methods section).

### The methylmalonyl- and ethylmalonyl-CoA pathways

**Fig 2:**
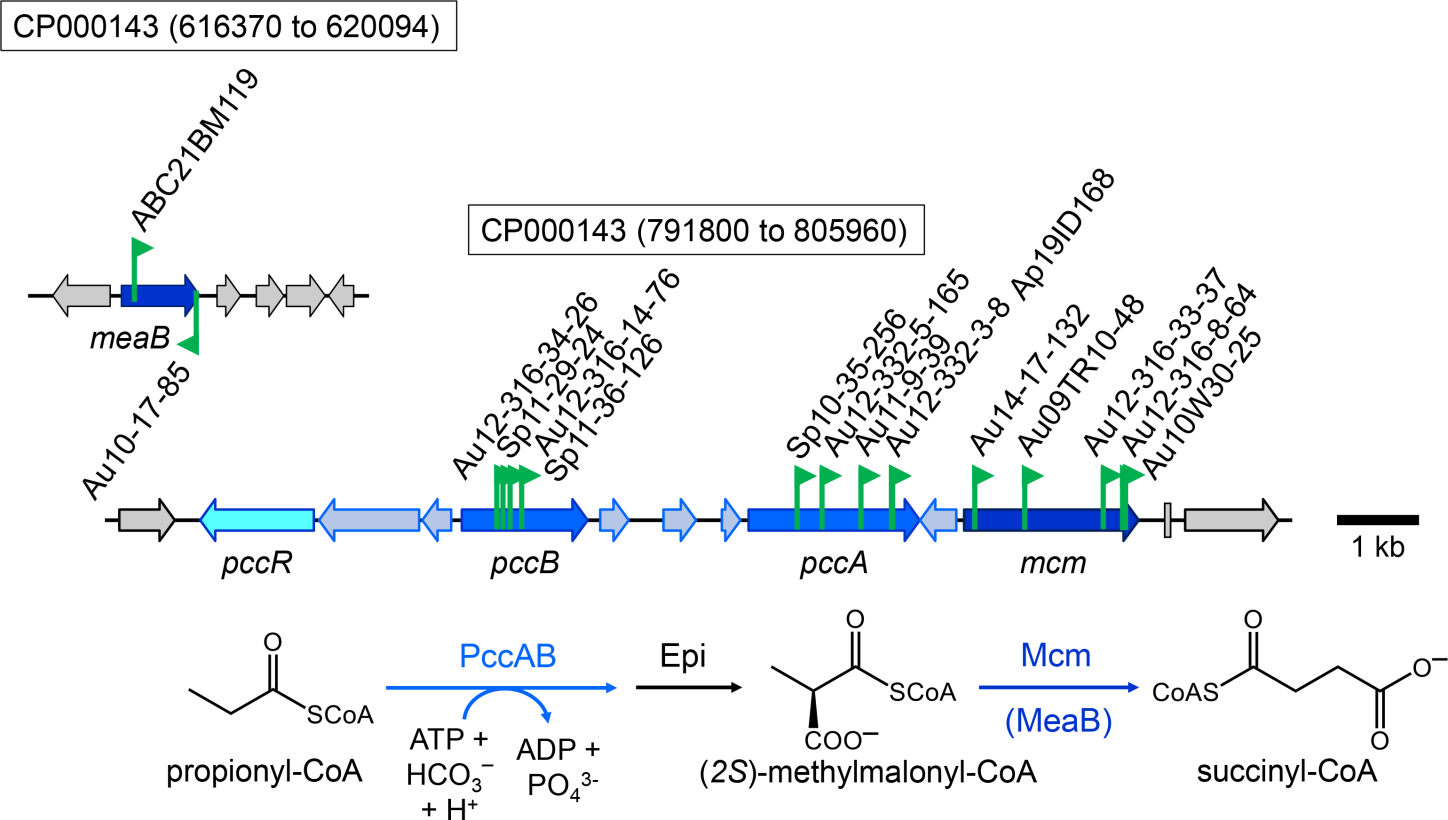
Map of transposon insertion sites at two genomic loci on chromosome 1 (accession number CP000143) encoding enzymes of the methylmalonyl-CoA pathway that converts propionyl-CoA and bicarbonate to succinyl-CoA. On plates, all *C. sphaeroides* transposon mutants were propionate/HCO_3_^–^ negative, butyrate/HCO_3_^–^ negative, and 3-hydroxypropionate compromised/negative on plates incubated either aerobically in the dark or anaerobically in the light. Mutants for which the transposon insertion mapped to *meaB* or *mcm* were also acetate negative, whereas all other mutants were only acetate compromised. Compromised growth is defined as significantly less growth compared to the growth of other mutants on the same plate. Flags mark the insertion site for each mutant, and the arrow indicates the transcriptional direction of the kanamycin resistance marker gene that is part of the transposon. PccB (accession number WP_002719342) and PccA are subunits of the propionyl-CoA carboxylase, and Mcm represents the methylmalonyl-CoA mutase. PccR is a ScfR-type transcriptional regulator controlling expression of *pccB* and possible downstream genes (9). MeaB (accession number WP_011337157) is required for methylmalonyl-CoA mutase maturation (14).

Mutants for which the transposon insertion site mapped to the gene cluster encoding enzymes of the methylmalonyl-CoA pathway were isolated as 3-hydroxypropionate-negative and acetate-negative or acetate-compromised on plates incubated aerobically (Table S1; Fig. 2). These mutants were later found to also be propionate/HCO_3_^–^- and butyrate/HCO_3_^–^- negative. The inability of the mutants to grow with propionate/HCO_3_^–^ is consistent with the proposal that the methylmalonyl-CoA pathway is the only propionyl-CoA assimilation pathway for *C. sphaeroides* (9). No growth with butyrate/HCO_3_^–^ and 3-hydroxypropionate suggests that propionyl-CoA is an intermediate in the metabolism of these carbon substrates. For 3-hydroxypropionate, propionyl-CoA has been shown to be a product of acrylyl-CoA reductase as part of the so-called reductive route for 3-hydroxypropionate assimilation by *C. sphaeroides* (6; Fig. 1). As expected, mutants for which the transposon mapped to the *acuI* gene were 3-hydroxypropionate-compromised during aerobic growth and 3-hydroxypropionate-negative during phototrophic growth in the light (Table S1), consistent with the previously characterized deletion/insertion mutant (6). Propionyl-CoA is also an intermediate in the assimilation of acetyl-CoA; however, *pcc* mutants were only acetate-growth compromised. Methylmalyl-CoA, formed from two acetyl-CoA and CO_2_, is cleaved into glyoxylate and propionyl-CoA in the ethylmalonyl-CoA pathway (Fig. 1). In principle, growth may proceed with acetate in the absence of propionyl-CoA carboxylase by forming malyl-CoA/malate from glyoxylate and acetyl-CoA. Because of the difference in the growth phenotypes for the *pcc* and *mcm* mutants with acetate, however, the possibility exists that propionyl-CoA, but not methylmalonyl-CoA, may be hydrolyzed and secreted as the free acid.

Apparently, the absence of a functional MeaB protein in the cell leads to an acetate-, butyrate/HCO_3_^–^-, and 3-hydroxypropionate-negative phenotype (Table S1; Fig. 2). MeaB from *Methylobacterium extorquens* was shown to be required to produce a functional coenzyme B_12_-dependent methylmalonyl-CoA mutase (Mcm) based on genetic evidence (15). Subsequent biochemical characterization revealed a role of MeaB as a chaperone, facilitating the insertion of the B_12_-cofactor into Mcm (14). Therefore, it was expected that the phenotype of the transposon mutants affecting *meaB* expression would mimic that of the transposon mutants for *mcm*, and this was confirmed (Table S1). MeaB may also assist in inserting the B_12_-cofactor into ethylmalonyl-CoA mutase; however, because both enzymes, methyl- and ethylmalonyl-CoA mutases, are needed for growth with acetate, a role of MeaB for the ethylmalonyl-CoA mutase cannot be tested using mutant studies.

Three other genes that encode enzymes catalyzing steps in the ethylmalonyl-CoA pathway, β-ketothiolase (PhaA), 3-hydroxybutyryl-CoA reductase (PhaB), and mesaconyl-CoA hydratase (Mch) were isolated as acetate-negative mutants (Table S1), consistent with the role of the ethylmalonyl-CoA pathway in connecting acetyl-CoA and succinyl-CoA/L-malate pools in central carbon metabolism (Fig. 1).

### Other acetate-compromised mutants

**Fig 3:**
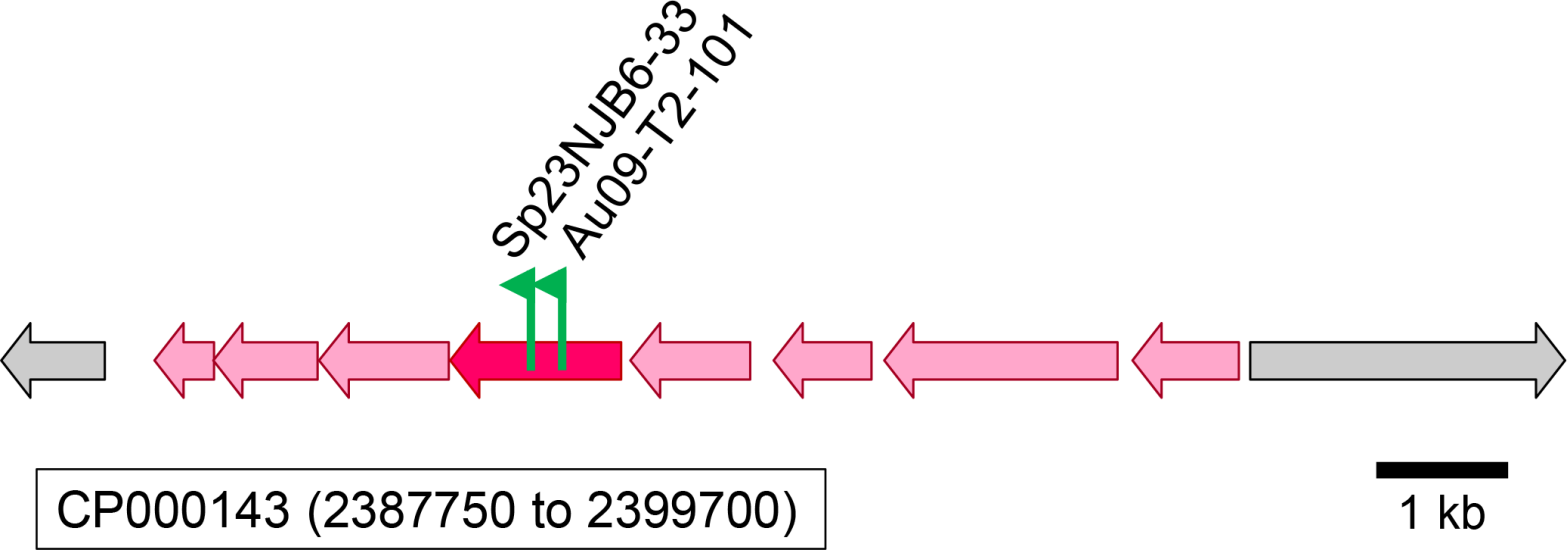
Transposon insertion sites for two mutants that had acetate-compromised growth on aerobically incubated plates but showed wild-type growth with succinate. Flags mark the insertion site for both mutants, and the arrow indicates the transcriptional direction of the kanamycin resistance marker gene that is part of the transposon. The gene interrupted (*rsp_0653*) likely encodes an UDP-glucose-6-dehydrogenase (accession number ABA79827.1). Downstream genes encode a possible UDP-glucose-4-epimerase, a protein related to inositol monophosphate phosphatase, and a secreted lipoprotein likely acting as a transglycosylase. Upstream genes encode transport systems.

Two mutants were isolated that were compromised for growth with acetate (vs succinate) on aerobically incubated plates (Table S1), but their gene products have no apparent function in the ethylmalonyl-CoA pathway. In both cases, the transposon interrupted the coding region for Rsp_0653, a protein belonging to the COG1004 family of UDP-glucose-6-dehydrogenase (*e*-value of 0). Rsp_0653 is 60% identical to the RkpK protein from *Sinorhizobium meliloti* (accession number WP_127540063) for which UDP-glucose dehydrogenase activity has been reported (16). Nucleotide-linked sugars are used for the synthesis of intracellular polysaccharides, such as starch or glycogen, or are targeted to the periplasm or the extracellular space for glycosyltransferase reactions. Upstream genes of *rsp_0653* encode transport systems, and downstream genes encode a transglycosylase that contains a signal peptide for secretion, and a UDP-glucose-4-epimerase (Fig. 3). Because of the annotation of Rsp_0653 and the genomic context of its gene, the Rsp_0653 protein is proposed to function in the modification of extracytoplasmic polysaccharides, and the mutants may, therefore, have a defect in cell envelope biogenesis; however, the reason for the effect on acetate-dependent, but not succinate-dependent, growth is unknown.

### An oxidative route for 3-hydroxypropionate metabolism

**Fig 4:**
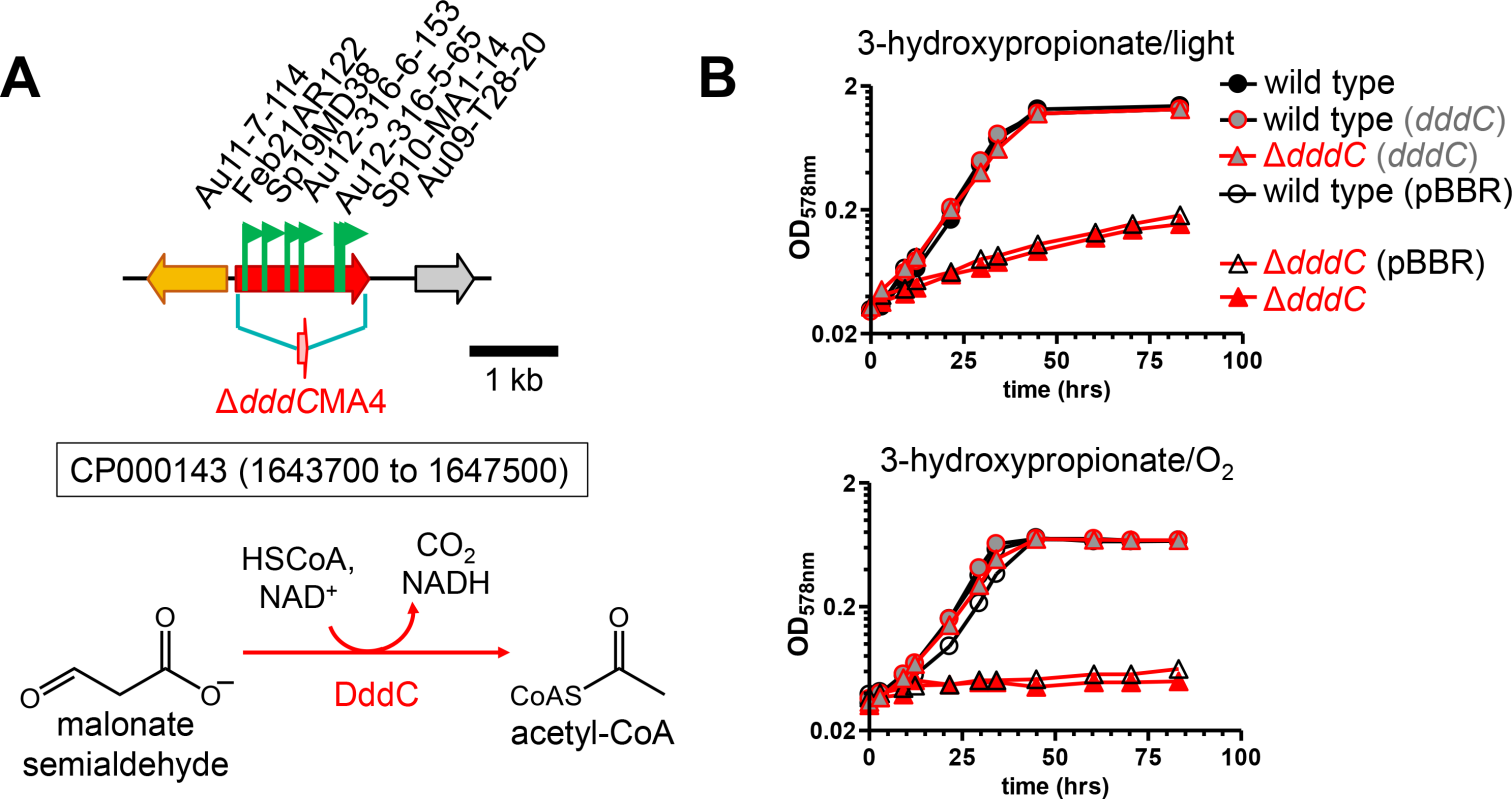
(**A**) Transposon insertion sites on chromosome 1 (accession number CP000143) mapping to the *dddC (rsp_2962*) gene proposed to encode an acetylating malonate semialdehyde dehydrogenase from *C. sphaeroides* (accession number ABA79124). Based on visual inspection, all transposon mutants listed were scored as 3-hydroxypropionate-negative on plates incubated either aerobically in the dark or anaerobically in the light. Flags mark the insertion site for each mutant, and the arrow indicates the transcriptional direction of the kanamycin resistance marker gene that is part of the transposon. For the Δ*dddC*MA4 strain, most of the coding region of *dddC* was deleted, with the in-frame deletion resulting in a 19-amino acid peptide, as indicated. The gene upstream of *dddC* and marked in orange encodes a LysR-type transcriptional regulator that may control the expression of *dddC*. (**B**) Growth of the wild-type and Δ*dddC*MA strains, carrying no plasmid, an empty vector control (pBBR) or the intact *dddC* gene on a pBBR-derived plasmid (*dddC*). Growth was either anaerobically in the light (photoheterotrophically, top graph) or aerobically in the dark (respiratory growth, bottom graph) with 3-hydroxypropionate as the carbon substrate. The same symbols for strains are used for both growth conditions.

A set of mutants for which the transposon insertion sites mapped to the *dddC* gene (*rsp_2962*) was isolated as 3-hydroxypropionate negative (Table S1; Fig. 4). For further characterization, an in-frame deletion mutant Δ*dddC*MA4 was generated that encodes a 19-amino acid peptide instead of the full-length DddC protein. The Δ*dddC*MA4 mutant was severely affected for photoheterotrophic growth with 3-hydroxypropionate, and aerobic respiratory growth with 3-hydroxypropionate was abolished, but growth was restored by introducing an intact *dddC* gene on a plasmid, whereas an empty plasmid control did not restore growth (Fig. 4B), confirming that *dddC* is required for 3-hydroxypropionate-dependent growth. DddC (accession number ABA79124) belongs to the family of acetylating methylmalonic acid semialdehyde dehydrogenases (protein family TIGR01722). The amino acid sequence for the full-length DddC protein is 42% identical to the biochemically characterized IolA protein from *Bacillus subtilis* ([17]; accession number AB005554.1) that functions as an acetylating malonate semialdehyde dehydrogenase in the metabolism of *myo*-inositol (18). Because the oxidation of 3-hydroxypropionate would lead to malonate semialdehyde and based on the sequence analysis, it is concluded that DddC represents a malonate semialdehyde dehydrogenase of *C. sphaeroides*.

**Fig 5:**
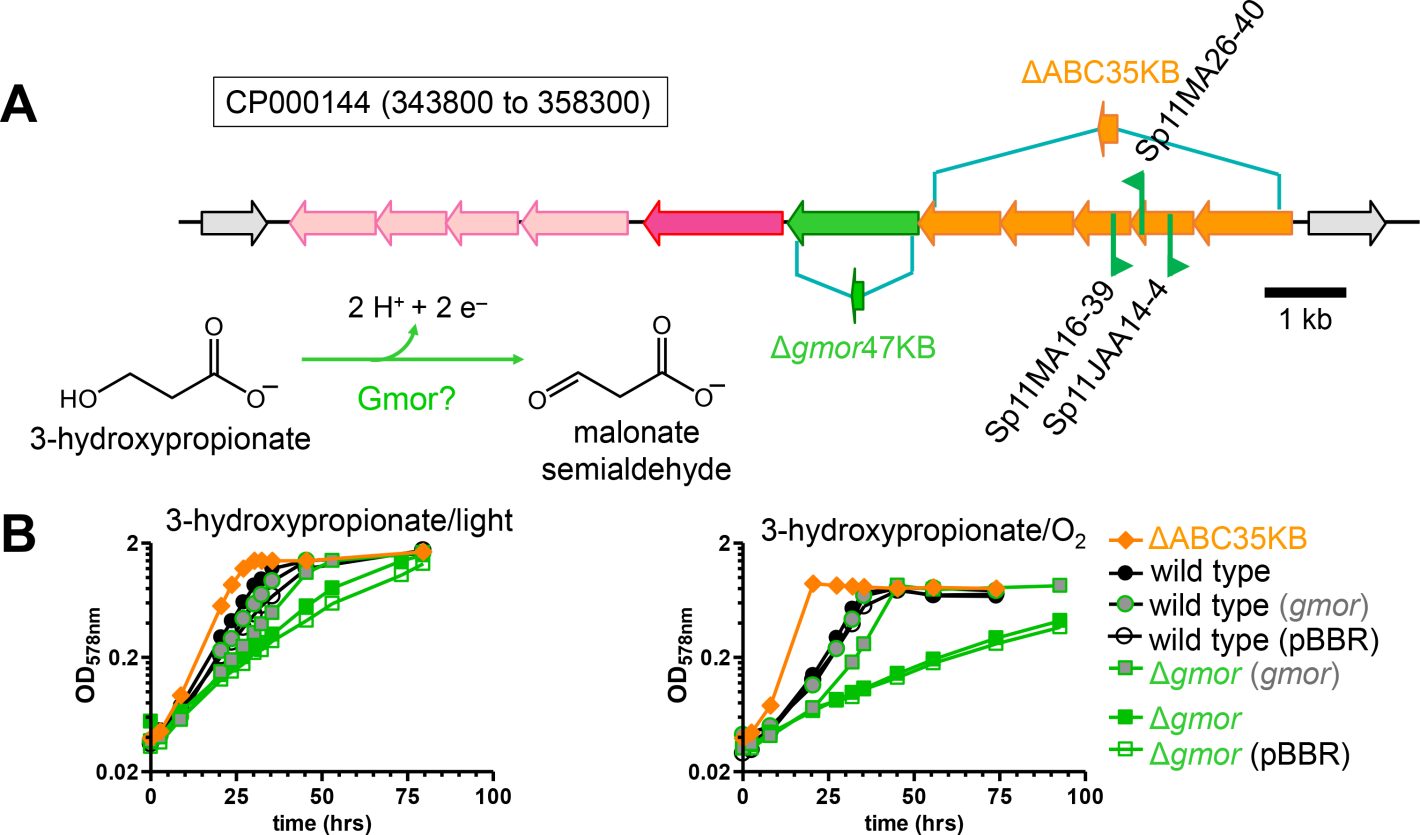
(**A**) Map of insertion sites on chromosome 2 (accession number CP000144) for transposon mutants of *C. sphaeroides* isolated on plates incubated aerobically with severely compromised 3-hydroxy-propionate-dependent growth. Flags mark the insertion site for each mutant, and the arrow indicates the transcriptional direction of the kanamycin resistance marker gene that is part of the transposon. For the ΔABC35KB mutant, most of the coding regions of five genes encoding an ABC transporter (orange) were deleted, resulting in a peptide consisting of 47 N-terminal amino acids of RSP_3297 (accession number ABA80904) and 44 C-terminal amino acids of RSP_3293, as indicated. For the Δ*gmor*47KB mutant, most of the coding region of *gmor* was deleted, with the in-frame deletion resulting in a 62-amino acid peptide, as indicated. The gene *rsp_3291*, shown in dark pink, is predicted to encode a symporter, and the four genes downstream of it, shown in light pink, encode an ABC transporter. (**B**) Growth of the wild-type and the Δ*gmor*47KB strains, carrying no plasmid, an empty vector control (pBBR) or the intact *gmor* gene on a pBBR-derived plasmid (*gmor*), as well as growth of the ΔABC35KB strain either anaerobically in the light (photoheterotrophically, left graph) or aerobically in the dark (respiratory growth, right) with 3-hydroxypropionate as the carbon source. Gmor (accession number ABA80899) is proposed to encode a 3-hydroxypropionate dehydrogenase.

Three transposon mutants of *C. sphaeroides* were isolated as either 3-hydroxypropionate negative or 3-hydroxypropionate compromised aerobically on plates and the transposon insertion sites mapped to genes encoding the two ATPase subunits of an ATP binding cassette (ABC) transporter (Table S1; Fig. 5). However, upon deletion of most of the coding regions of all five genes encoding the ABC transporter, 3-hydroxypropionate-dependent growth of the ΔABC35KB mutant was observed, and aerobic respiratory growth of the ΔABC35KB mutant with 3-hydroxypropionate in three independent experiments (doubling time: 3.7 ± 0.3 hours) was even faster than the growth of the wild-type strain with 3-hydroxypropionate (doubling time: 6.5 ± 0.6 hours) under the same conditions. Only two nucleotides separate the *gmor* gene from the genes encoding the ABC transporter (Fig. 5A), and it can be assumed that these genes are co-transcribed, possibly also with *rsp_3291* annotated to encode a sodium/phosphate symporter (61 nucleotide spacing) and even further downstream genes encoding another ABC transporter, although with a 200 nucleotide spacing between *rsp_3291* and *rsp_3290*, the presence of another promoter is also possible. To test if the 3-hydroxypropionate diminished growth observed with the transposon mutants was due to a polar effect on *gmor*, the *gmor* gene was introduced on a plasmid into the SP11MA16-39 transposon mutant. Indeed, the introduction of the *gmor* gene under a constitutive promoter restored growth of the Sp11MA16-39 mutant with 3-hydroxypropionate as the carbon source (Fig. S1), and it was, therefore, concluded that the initial 3-hydroxypropionate-compromised phenotype was due to the lack of Gmor in the cell, rather than due to the lack of the ABC transporter. An in-frame deletion mutant Δ*gmor*47KB was obtained that encodes a 62-amino acid peptide instead of the full-length Gmor protein. The Δ*gmor*47KB mutant displayed a growth defect for photoheterotrophic growth with 3-hydroxypropionate, and the slower growth of the mutant compared to wild type was more pronounced during aerobic respiratory growth with 3-hydroxypropionate (Fig. 5B). Introduction of an intact *gmor* gene on a plasmid restored growth, whereas an empty plasmid control did not, confirming that *gmor* is required for optimal growth with 3-hydroxypropionate. Gmor belongs to the superfamily of flavin-containing glucose-methanol-choline oxidoreductases (Pfam00732, [19]). Choline dehydrogenase oxidizes the alcohol group of choline and forms betaine aldehyde. Instead of the trimethylammonium moiety in choline as the electron-withdrawing group for the oxidation of the activated alcohol, 3-hydroxypropionate has a carboxylic acid group, and we, therefore, proposed that Gmor oxidizes 3-hydroxypropionate to its aldehyde, malonate semialdehyde. Gmor would then function together with DddC in oxidizing 3-hydroxypropionate to acetyl-CoA and CO_2_. An oxidative route for 3-hydroxypropionate conversion to acetyl-CoA via malonate semialdehyde was also proposed for *Halomonas* sp. HTNK1 (20). For this marine bacterium, 3-hydroxypropionate is an intermediate in the metabolism of dimethylsulfoniopropionate (DMSP), and *dddC*- and *gmor*-like genes are adjacent in a gene cluster encoding other enzymes required for the metabolism of DMSP (Fig. S2), supporting the assigned function of Gmor as a 3-hydroxypropionate dehydrogenase and DddC as a malonate semialdehyde dehydrogenase. In a metabolic context, it can be predicted that in the presence of an oxidative route for the conversion of 3-hydroxypropionate to acetyl-CoA and CO_2_, pyruvate dehydrogenase would be dispensable during 3-hydroxypropionate-dependent growth (Fig. 1); and this was confirmed (see below).

### Pyruvate dehydrogenase

**Fig 6:**
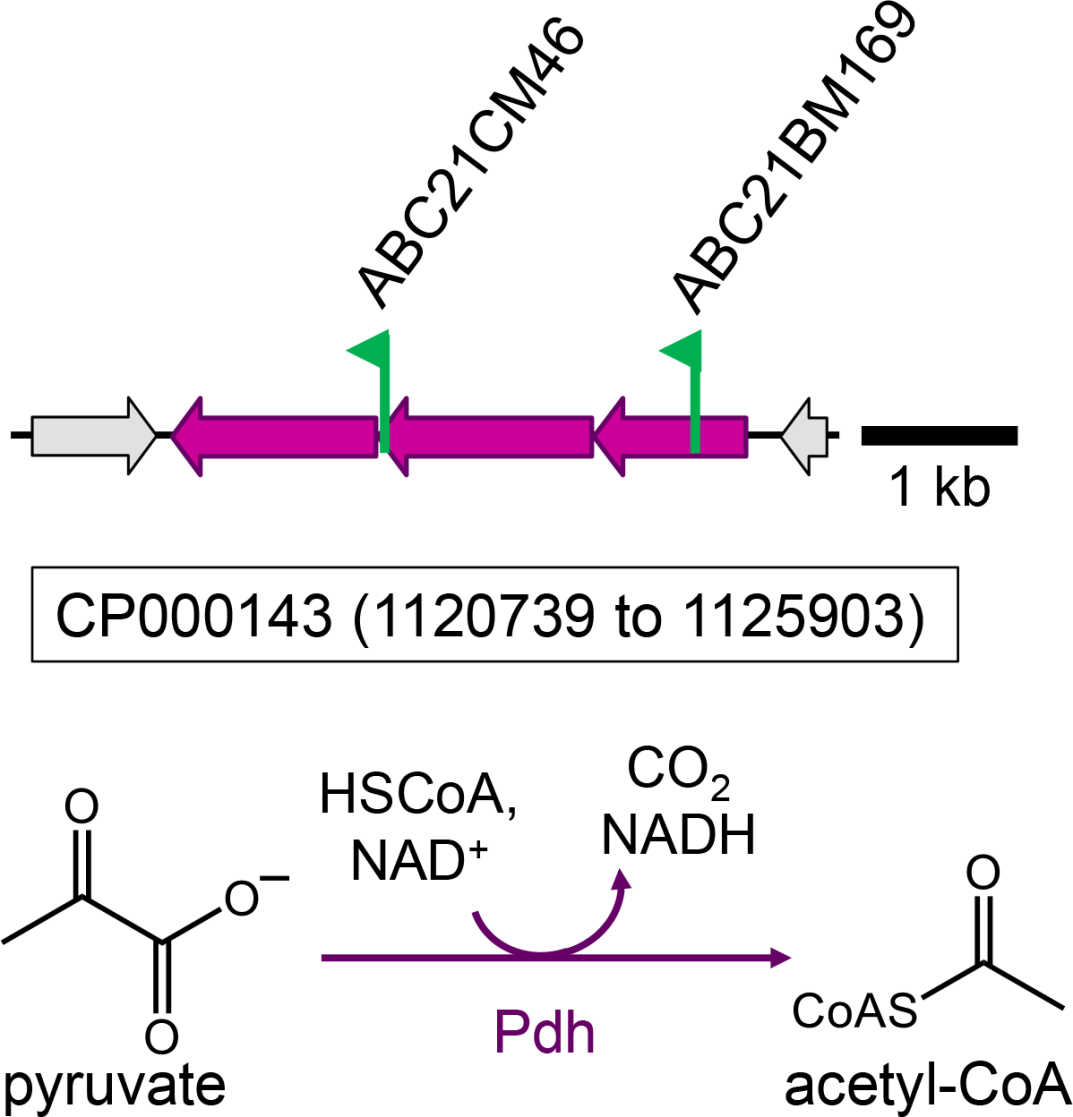
Transposon insertion sites on chromosome 1 (accession number CP000143) mapping to two genes (*rsp_4047* and *rsp_4049*) proposed to encode the alpha- (accession number ABA78645.1) and beta-subunits of the E1 component of pyruvate dehydrogenase (Pdh) from *C. sphaeroides*. The mutants were isolated as D-lactate, L-lactate, or L-malate-negative on plates incubated aerobically in the dark. A full range of substrates was not tested for these mutants; however, the mutants were acetate- and 3-hydroxy-propionate-positive on plates incubated aerobically in the dark. Flags mark the insertion site for each mutant, and the arrows indicate the transcriptional direction of the kanamycin resistance marker gene that is part of the transposon.

A transposon mutant library was constructed using acetate/HCO_3_^–^ as the carbon substrate, and this library was screened for aerobic growth with L-malate, 3-hydroxypropionate, L-lactate, and D-lactate. Two of the mutants identified only grew with 3-hydroxypropionate and the control substrate (acetate/HCO_3_^–^), and the transposon insertion site mapped to a possible three-gene operon predicted to encode the E1-component (pyruvate decarboxylase) and the two subunits of the E2-component (dihydrolipoamide acetyltransferase) of pyruvate dehydrogenase (Fig. 6). The third catalytic E3-component (dihydrolipoamide dehydrogenase) of the large pyruvate dehydrogenase complex may be shared with alpha-ketoglutarate dehydrogenase (Rsp_0962–Rsp_0966) or may be encoded by *rsp_2968*. Regardless, both transposon mutants, ABC21CM46 and ABC21BM169, are predicted to have lost pyruvate dehydrogenase activity because of their inability to grow with D-lactate, L-lactate, or L-malate. However, these mutants were able to use 3-hydroxypropionate as a carbon substrate, consistent with the presence of an oxidative route, forming acetyl-CoA (and CO_2_) directly from 3-hydroxypropionate, making pyruvate dehydrogenase dispensable during 3-hydroxypropionate-dependent growth.

### Pyruvate carboxylase

**Fig 7:**
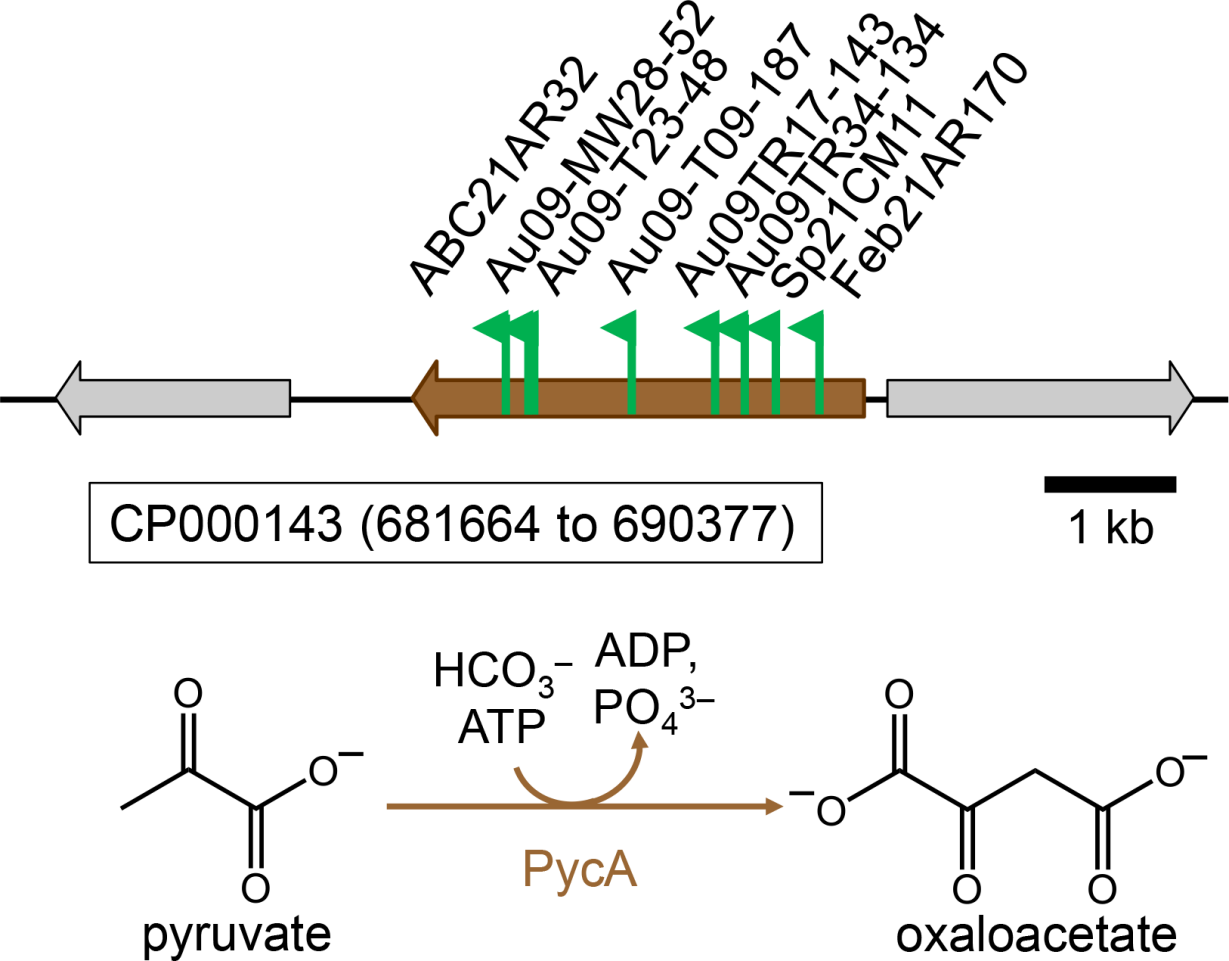
Transposon insertion sites on chromosome 1 (accession number CP000143) mapping to the *pycA* (*rsp_2090*) gene proposed to encode pyruvate carboxylase from *C. sphaeroides* (accession number ABA78245.1). All mutants listed were isolated as D-lactate-, L-lactate- D/L-lactate, or D-malate-negative, but L-malate-positive on plates incubated aerobically in the dark. Flags mark the insertion site for each mutant, and the arrows indicate the transcriptional direction of the kanamycin resistance marker gene that is part of the transposon.

A major control point for carbon distribution in the central carbon metabolism of any organism is the so-called PEP-pyruvate-oxaloacetate node that connects C3- and C4-containing central carbon intermediates (21). Several transposon mutants of *C. sphaeroides* were isolated that had lost the ability to use carbon substrates, such as lactate and D-malate, that are required to fill C4-precursor metabolite pools starting from C3-intermediates of central carbon metabolism and the transposon insertion sites mapped to the *pycA* (*rsp_2090*) gene (Table S1; Fig. 7). The amino acid sequence of PycA (accession number ABA78245) identifies it as a biotin-dependent pyruvate carboxylase (TIGR01235 protein family of pyruvate carboxylases, *e*-value = 0). The *rsp_2090* gene product has 52% sequence identity with the pyruvate carboxylase from *Corynebacterium glutamicum* (accession number WP_254503367) that has been characterized (22). A gene candidate for a PEP carboxylase was not identified in the genome of *C. sphaeroides*, and in a previous study, only pyruvate carboxylase and no PEP carboxylase activity was detected in cell extracts of *C. sphaeroides,* and pyruvate carboxylase-deficient mutants had lost the ability to grow with glucose or pyruvate as a carbon source (23). Similarly, for *Rhodobacter capsulatus*, a close relative of *C. sphaeroides*, the pyruvate carboxylase protein and its activity were upregulated during growth with lactate, fructose, glucose, pyruvate, and D-malate, but were downregulated during growth with carbon substrates like α-ketoglutarate, L-malate, or succinate, and pyruvate carboxylase-deficient mutants had lost the ability to grow with pyruvate or lactate (24, 25). These results are consistent with the conclusion that pyruvate carboxylase is the enzyme that connects the C3- and C4-precursor metabolite pools as part of the PEP-pyruvate-oxaloacetate node for *C. sphaeroides* (and for *R. capsulatus*).

### Transporters

Automated annotation predicts that the genome of *C. sphaeroides* encodes many ABC transporters in addition to several TRAP transporters (26). Both types of transport systems rely on an extracytoplasmic solute-binding protein. The founding member of TRAP transporters that lack an ATP-binding/hydrolyzing component is the transport system for C4-dicarboxylic acids (Dct) that was discovered using a transposon mutagenesis screen with *R. capsulatus* (27, 28). This transport system is not related to the transport system for C4-dicarboxylic acids (DctA) of *Escherichia coli* (29). In this study, transposon mutants isolated as L-malate and succinate-negative were recovered and the insertion sites mapped to *rsp_0910* and *rsp_0912*, encoding proteins (accession numbers ABA80092.1 or ABA80094.1) with 89% or 86% amino acid sequence identity to the periplasmic substrate-binding protein component (DctP) or to one of the two membrane components (DctM) of the Dct-system from *R. capsulatus*, respectively (Table S1), consistent with a similar system functioning in C4-dicarboxylic acid uptake for *C. sphaeroides*.

**Fig 8:**
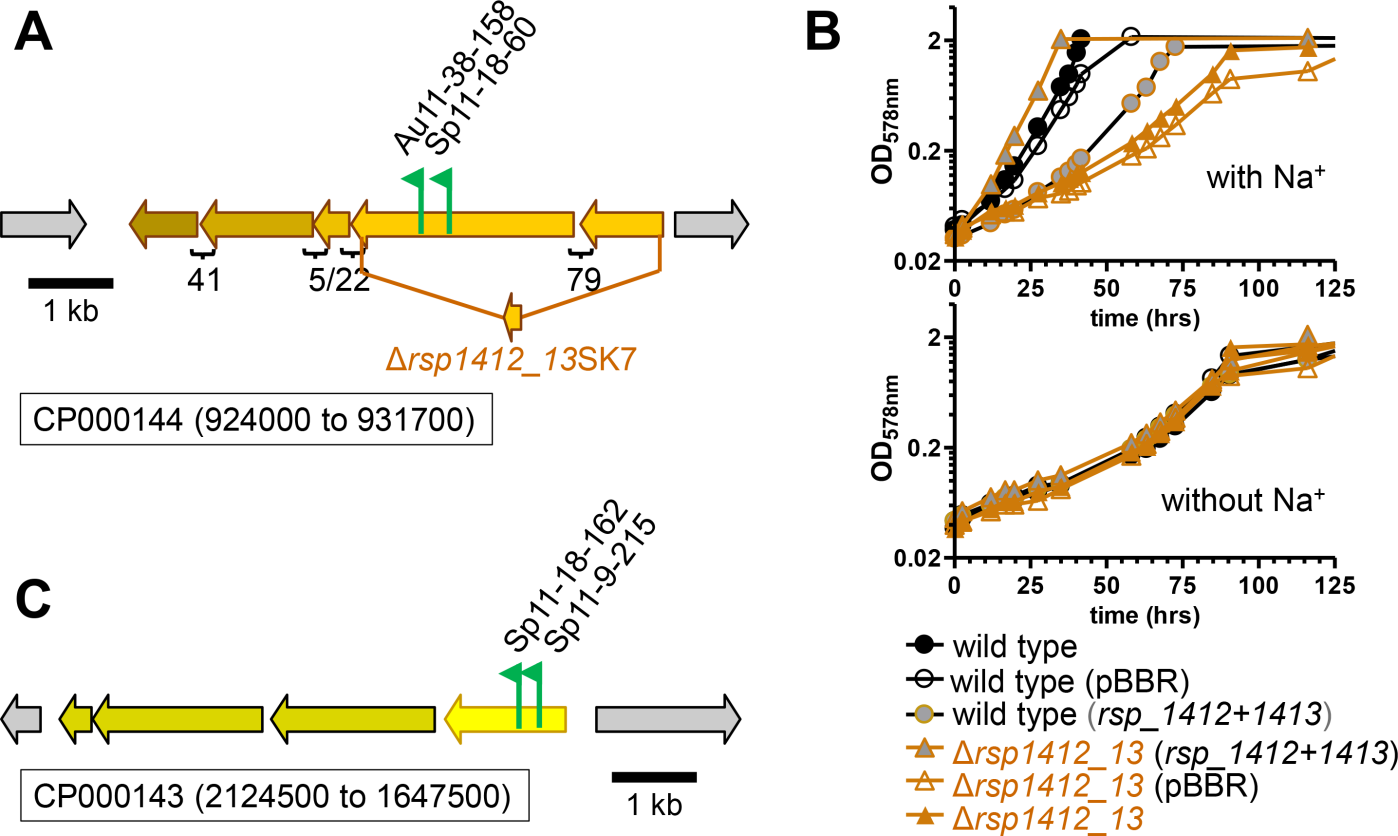
L-Glutamate-negative or -compromised mutants. Insertion sites for transposon mutants isolated as L-glutamate negative (**A**) or L-glutamate compromised (**C**) on plates incubated aerobically in the dark. Flags mark the insertion site for each mutant, and the arrows indicate the transcriptional direction of the kanamycin resistance marker gene that is part of the transposon. (**A**) For the possible operon on chromosome 2 (CP000144), the first two genes (*rsp_1412* and *rsp_1413*) encode the periplasmic-binding protein (Rsp_1412, accession number AMJ49705.1) and the membrane component (Rsp_1413, accession number: AMJ49704.1) of a TRAP transporter from *C. sphaeroides*, respectively. The three genes (*rsp_3804-3802*) downstream are likely co-transcribed with *rsp_1412* and *rsp_1413* because of the short spacing between the open reading frames, as indicated. (**B**) Phototrophic growth of the wild-type and the Δ*rsp_1412-1413*SK7 deletion strains, carrying no plasmid, an empty vector control (pBBR) or the intact *rsp_1412* and *rsp_1413* genes on a pBBR-derived plasmid (*rsp_1412 + 1413*), with L-glutamate as the carbon source, either in the presence (top) or absence (bottom graph) of sodium ions in the medium. The genes *rsp_1412* and *rsp_1413* are likely to encode a sodium-dependent TRAP transport system that was previously biochemically characterized by Jacobs et al. (12). (**C**) Transposon insertion sites on chromosome 1 (CP000143) mapping to the *rsp_0398* gene proposed to encode a glutamate dehydrogenase from *C. sphaeroides* (accession number ABA79572.1) for mutants that were isolated as L-glutamate compromised on plates.

For two L-glutamate negative mutants of *C. sphaeroides*, the transposon insertion sites mapped to a gene encoding a component of another TRAP transporter, specifically of the TAXI family (protein family TIGR02122; Table S1; Fig. 8A). The substrate specificity of the TRAP transporter cannot be predicted by the protein sequence, but the extracytoplasmic substrate binding (accession number: ABA81414.1) belongs to the family of TAXI-TRAP transporters with a proposed glutamate/glutamine binding protein from *Thermus thermophilus* as one of its members (30). Because of the L-glutamate negative plate phenotype, it is proposed that *rsp_1412* and *rsp_1413* encode a TRAP transporter for L-glutamate. The short spacing between open reading frames (5–41 bp) suggests that the three genes (*rsp_3804-3802*) downstream are co-transcribed with *rsp_1412* and *rsp_1413*, and their gene products are likely functionally related to L-glutamate uptake. Almost 30 years ago, two L-glutamate transport systems for *C. sphaeroides* were biochemically characterized by Jacobs et al. (12, 31, 32). Both transport systems were dependent on extracytoplasmic binding proteins, but one of the two transporters was energized not by ATP hydrolysis (ABC transporter) but was relying on the electrochemical gradient. This secondary transport system for L-glutamate was shown to be sodium ion-dependent, and the authors recognized it, at the time, as a novel binding-protein-dependent transport system; however, the genes encoding the transport system were not identified (12). To test if the genes identified here by the transposon mutagenesis screen may encode this sodium-dependent TRAP transporter, an in-frame deletion mutant RsΔ*1412_13*SK7 was obtained that encodes a peptide consisting of a 9-amino acid N-terminal sequence of Rsp_1413 and a 29-amino acid C-terminal sequence of Rsp_1412 with three additional amino acids at the junction (Fig. 8A). When tested for L-glutamate-dependent growth in the presence or absence of sodium ions, the deletion mutant had lost its sodium-dependent growth with L-glutamate (Fig. 8B). Introduction of both intact *rsp_1412* and *rsp_1413* genes on a plasmid fully restored L-glutamate-dependent growth in the presence of sodium ions, whereas an empty plasmid control did not, confirming that *rsp_1412* and *rsp_1413* are required for sodium-dependent enhancement of growth with L-glutamate. Remaining growth of the RsΔ*1412_13*SK7 is probably due to the sodium-independent ABC transport system for L-glutamate (31). These results are consistent with *rsp_1412* and *rsp_1413* encoding the sodium-dependent TRAP transporter for L-glutamate of *C. sphaeroides* that was previously characterized biochemically but for which the genes were not identified at the time (12).

### L-Glutamate metabolism

In addition to the L-glutamate transport-deficient mutants, two L-glutamate-compromised mutants of *C. sphaeroides* were isolated, and the transposon insertion sites mapped to *rsp_0398* on chromosome 1 (Table S1; Fig. 8C). The protein RSP_0398 belongs to the family of glutamate/leucine/phenylalanine/valine dehydrogenases (COG0334). Because of the L-glutamate compromised phenotype, it is proposed that RSP_0398 can use glutamate as a substrate and oxidizes/deaminates it to enter central carbon metabolism at the level of alpha-ketoglutarate. However, because the mutants are still able to grow with L-glutamate as the only carbon source, there may be another enzyme that at least partially fulfills the same or similar function for *C. sphaeroides*.

### Pigmentation mutants

In addition to the isolation of transposon mutants that had lost the ability to use specific carbon substrates, several strains were analyzed because they displayed a colony color distinct from wild type (Table S1). Most of these pigmentation mutants had a transposon insertion in one of several genes, clustered at a 45 kbp locus, encoding enzymes for carotenoid and bacteriochlorophyll synthesis (Fig. S3) and not surprisingly, most of these mutants had lost the ability to grow in the light. Other phototrophic-negative mutants forming white colonies had interruptions in genes encoding the regulatory proteins AppA and PrrA (RegA; (Table S1; Fig. S4). A group of transposon mutants sporadically appeared as pale colonies on plates with acetate or 3-hydroxypropionate as the carbon source and the transposons mapped to the *rsp_1670* gene that encodes a RelA/SpoT homolog as part of the stringent response (Table S1; Fig. S5). For more information on these mutants, please refer to the supplementary material section.

## DISCUSSION

A forward genetic screen led to the isolation of *C. sphaeroides* transposon mutants that have lost the ability to use one or more of the following carbon sources: 3-hydroxypropionate, D-malate, L-malate, propionate/HCO_3_^–^, butyrate/HCO_3_, L-lactate, D-lactate, acetate, and L-glutamate. These carbon substrates only require a few steps to enter carbon metabolism, and most mutants recovered disrupted the core metabolism of *C. sphaeroides*, instead of affecting peripheral pathways unique to each carbon substrate. The elucidation of the ethylmalonyl-CoA pathway, as a reaction sequence connecting the two precursor metabolite pools of acetyl-CoA and oxaloacetate, was facilitated by the isolation of transposon mutants that had lost the ability to grow with methanol (*Methylobacterium extorquens* [33]) or acetate (*C. sphaeroides* [34]). For the present study, the emphasis was on recovering *C. sphaeroides* mutants affecting the metabolism of 3-hydroxypropionate, although carbon compounds other than 3-hydroxypropionate were included in the screen, but growth-deficient mutants were not always followed up.

It has been previously shown that for *C. sphaeroides*, 3-hydroxypropionate enters the central carbon metabolism at the level of succinyl-CoA (Fig. 1; 5 ). In this so-called reductive route, 3-hydroxypropionate is activated to its CoA-ester, and after dehydration of 3-hydroxypropionyl-CoA, acrylyl-CoA is reduced to form propionyl-CoA, catalyzed by an NADPH-dependent acrylyl-CoA reductase that has been biochemically characterized (6). It has been proposed that the subsequent ATP-dependent addition of bicarbonate to propionyl-CoA, catalyzed by a biotin-dependent propionyl-CoA carboxylase, forms (*2R*)-methylmalonyl-CoA. After epimerization, the carbon skeleton of (*2S*)-methylmalonyl-CoA is rearranged to form the central carbon intermediate succinyl-CoA. The interconversion of (*2S*)-methylmalonyl-CoA and succinyl-CoA is expected to be catalyzed by a corrinoid-containing methylmalonyl-CoA mutase. The requirement to convert propionyl-CoA and bicarbonate to succinyl-CoA during the metabolism of 3-hydroxypropionate by *C. sphaeroides* was confirmed in this study, because mutants with transposon insertions in genes encoding either subunits of the propionyl-CoA carboxylase or the gene encoding the methylmalonyl-CoA mutase were 3-hydroxypropionate negative (Fig. 2; Table S1). The MeaB chaperone protein was also shown to be essential during growth with 3-hydroxypropionate, consistent with the fact that it is required for the maturation of the methylmalonyl-CoA mutase by facilitating the insertion of the B_12_-cofactor (14).

Genetic evidence provided in the current study points to acetyl-CoA as a second entry point of 3-hydroxypropionate into central carbon metabolism. In this oxidative route, 3-hydroxypropionate is oxidized to malonate semialdehyde and further to acetyl-CoA and CO_2_. Gene candidates encoding a 3-hydroxypropionate dehydrogenase (Gmor, accession number ABA80899) and an acetylating malonate semialdehyde dehydrogenase (DddC, accession number ABA79124) were identified (Fig. 4 and 5; Table S1). The requirement for an oxidative route is supported by the fact that pyruvate dehydrogenase was not required for growth with 3-hydroxypropionate (Fig. 6; Table S1), because in the absence of the oxidative route, acetyl-CoA would need to be formed via succinyl-CoA and via pyruvate (Fig. 1). Still, we cannot rule out that residual growth of the 3-hydroxypropionate dehydrogenase mutant (Δ*gmor*47KB) is due to only the reductive route functioning, because an intact pyruvate dehydrogenase is produced. However, a direct conversion of 3-hydroxypropionate to the precursor metabolite acetyl-CoA decreases the amounts of transiently produced CO_2_/HCO_3_^–^ and thereby limits the possibility for the loss of inorganic carbon as an electron acceptor from the cell that would lead to a redox imbalance during phototrophic growth (11). Residual growth in the absence of the 3-hydroxypropionate dehydrogenase (Δ*gmor*47KB) may, therefore, also be explained due to the activity of another enzyme partially fulfilling its role but at the same time, making 3-hydroxypropionate to acetyl-CoA/CO_2_ conversion overall growth rate-limiting. It is impossible to predict which enzyme may be responsible for this residual 3-hydroxypropionate oxidizing activity, because enzyme candidates can belong to many different dehydrogenase superfamilies. During aerobic respiratory growth, the oxidative route, together with the citric acid cycle, allows for the complete oxidation of 3-hydroxypropionate to CO_2_. The higher flux demand during aerobic growth through the oxidative route may be the reason why the absence of malonate semialdehyde dehydrogenase or 3-hydroxypropionate dehydrogenase resulted in a more severe growth defect during aerobic respiratory growth compared to phototrophic growth (Fig. 4).

Based on the analysis of mutants together with biochemical studies on the acrylyl-CoA reductase, the paths that connect the carbon of 3-hydroxypropionate with all precursor metabolite pools seem to now be complete: 3-hydroxypropionate has two entry points into central carbon metabolism, a reductive route converts the three carbons of this carbon source together with bicarbonate to succinyl-CoA, and an oxidative route that converts 3-hydroxypropionate to acetyl-CoA and CO_2_. All other precursor metabolites can then be formed from succinyl-CoA and acetyl-CoA by known routes (Fig. 1). Precursor metabolites donate their carbon to biosynthesis pathways leading to building blocks, cofactors, and secondary metabolites; however, the relative amounts of precursor metabolites to make a cell need to be considered as well, and these parameters are the basis for metabolic flux balance analysis (35). The paths connecting the various precursor metabolite pools involve carboxylation and decarboxylation reactions and, therefore, the relative flux through these pathways results in either net CO_2_ release or net inorganic carbon fixation (Fig. 1). The requirement for net release of inorganic carbon during growth is determined by the average oxidation state of the carbon of the growth substrate; in the case of 3-hydroxypropionate, it was determined that about 8% of the total carbon was released as CO_2_ with the remaining carbon being assimilated during phototrophic growth, consistent with theoretical predictions (11). Consequently, carbon flow through the ethylmalonyl-CoA pathway during growth with 3-hydroxypropionate is also required to ensure that the appropriate amount of inorganic carbon is released from the cell to achieve an overall redox balance (11).

For a transposon mutagenesis experiment as described here, using a defined medium, one can expect about 600 genes to be essential, as their products provide housekeeping functions or encode enzymes of biosynthetic pathways (36). In addition, in the current study, only a few carbon substrates during aerobic growth were tested. Nonetheless, random mutagenesis and screening of mutants allows the prediction of substrate specificity for protein families for which only general functions can be assigned bioinformatically, such as transporters. During a similar transposon mutagenesis screen with *R. capsulatus*, mutants were recovered that had lost the ability to use malate or succinate as a carbon substrate, and it was then shown that the inactivated genes encoded for a new type of extracytoplasmic solute-binding protein-dependent secondary transport system (27, 28). These so-called TRAP systems have now been recognized to be widespread in the *Bacteria* and *Archaea* domains (37). The substrate specificities of several clades of the TRAP transporter superfamily were determined using a high-throughput screen testing if potential ligands increased, upon binding, the unfolding transition temperature of purified solute-binding proteins by differential scanning calorimetry (38). However, although L-glutamate was included as a possible ligand, no TRAP transporter candidate for L-glutamate was identified. We propose that the genes *rsp_1412* and *rsp_1413* of *C. sphaeroides* encode the cytoplasmic membrane permease (AMJ49704.1) and the extracytoplasmic solute-binding protein component (AMJ49705.1) of a sodium-dependent L-glutamate uptake system, because the RsΔ*1412_13*SK7 deletion mutant had lost its sodium-dependent growth with L-glutamate (Fig. 8). We further propose that this transport system, which belongs to the TRAP-associated extracytoplasmic immunogenic (TAXI)-type (protein family TIGR02122), is identical with the sodium-dependent L-glutamate secondary transport system biochemically characterized by Jacobs et al., who at that time did not identify the corresponding genes (12). The other TRAP transporter identified in the current study is the DctPQM system previously described to transport L-malate and succinate, consistent with our finding (Table S1, ref. 28). The DctP extracytoplasmic solute-binding protein component of *R. capsulatus* is considered the founding member of the DctP-type TRAP transporters (protein family TIGR00787).

Once a carbon substrate is taken up and converted to a central carbon intermediate, all precursor metabolites need to be synthesized. Different enzymes connect these precursor metabolite pools depending on the entry point of the substrate. The carbon substrates L- and D-lactate and D-malate enter central carbon metabolism at the level of pyruvate. The growth defect for these substrates in the absence of pyruvate carboxylase confirms earlier reports that suggest that pyruvate carboxylase is the enzyme connecting the precursor metabolite pyruvate with oxaloacetate for *C. sphaeroides* (Fig. 7, [ 23]). Starting with pyruvate, acetyl-CoA is formed via pyruvate dehydrogenase, and therefore, the enzyme is needed for growth with L- and D-lactate, and L-malate (Fig. 6), and is expected to be also needed for growth with succinate, L-glutamate, and propionate/HCO_3_^–^; however, this was not tested.

A collection of transposon mutants allows testing of independent mutants with insertion sites at the same locus for consistency of the phenotype, also with additional carbon substrates that were not included in the initial screen. Many carbon substrates for *C. sphaeroides* are not known, and in the future, the use of central carbon metabolism mutants will allow proposing entry points for these carbon sources into the central carbon metabolism because of the distinct requirement of reactions interconverting precursor metabolites dependent on the entry point into the central carbon metabolism for a group of substrates. In the current study, it was established that 3-hydroxypropionate is initially converted into two central carbon intermediates, acetyl-CoA and succinyl-CoA. Either one entry point into central carbon metabolism would have been satisfactory to explain 3-hydroxypropionate assimilation, and having an oxidative route together with the ethylmalonyl-CoA pathway and a reductive route forming succinyl-CoA may seem redundant at first (Fig. 1). The fact, though, is that having both options reduces transiently produced CO_2_ and possibly limits the loss of inorganic carbon as an electron acceptor from the cell that would lead to a redox imbalance (11).

## MATERIALS AND METHODS

### Bacterial strains and growth conditions

*C. sphaeroides* (DSMZ 158, formerly *R. sphaeroides* 2.4.1) was grown either aerobically in the dark or anaerobically with continuous illumination by incandescent light bulbs (3,000 lux) at 25°C–29°C with minimal medium (MM) containing (per liter) 1.2 g NH_4_Cl, 0.2 g MgSO_4_·2H_2_O, 0.07 g CaCl_2_·2H_2_O, 15 mM potassium phosphate buffer, pH 6.7, 10 mL of a trace element solution (500 mg disodium EDTA, 300 mg FeSO_4_·7H_2_O, 3 mg MnCl_2_·4H_2_O, 50 mg CoCl_2_·6H_2_O, 1 mg CuCl_2_·2H_2_O, 2 mg NiCl_2_·6H_2_O, 3 mg Na_2_MoO_4_·2H_2_O, 5 mg ZnSO_4_·7H_2_O, and 2 mg boric acid per liter), and 1.5 mL of a vitamin solution (100 mg cyanocobalamin, 300 mg pyridoxamine dihydrochloride, 100 mg calcium pantothenate, 200 mg thiamine hydrochloride, 200 mg nicotinic acid, 80 mg 4-aminobenzoic acid, and 20 mg D-[+]-biotin per liter). The sodium salts of the following acids were used as carbon substrates at 10 mM: succinate, D/L-lactate, D-lactate, L-lactate, 3-hydroxypropionate, L-malate, D-malate, butyrate, propionate, bicarbonate, or acetate. If needed, kanamycin or spectinomycin was added to a final concentration of 20 µg mL^–1^ and 25 µg mL^–1^, respectively. For plates, the media contained 12.5% agar, and plates were incubated aerobically in the dark or anaerobically in the light. For aerobic liquid growth, a loose-capped 23 mL tube was filled with 4.5 mL of medium and shaken at 217 rpm. Anaerobic liquid growth was performed as previously described (11). *E. coli* strains were grown aerobically using Luria-Bertani (LB) broth at 37°C and, if needed, with ampicillin (100 µg mL^–1^), spectinomycin, or kanamycin (50 µg mL^–1^ each).

### Transposon mutagenesis

Wild-type *C. sphaeroides* and *E. coli* strain WM2672 (BW20767 [pRL27]; [13]) were grown aerobically with LB medium (25 mL cultures in 50 mL Erlenmeyer flasks) to an optical density of OD_578nm_ = 0.3–0.6, and the cells were collected by centrifugation at 8,000 × *g* at 4°C and washed twice with LB medium. Conjugation of *C. sphaeroides* and *E. coli* was performed at a ratio of 1:1 for 20–23 hours by placing a concentrated mixture of cells (est. OD_578nm_ = 35) on an LB agar plate and incubating it at 29°C in the dark (in air). The cells were scraped from the plates and resuspended in MM (typically containing succinate or D/L-lactate as the control carbon substrate), and 1:1 (undiluted), 1:10, and 1:100 dilutions were spread plated on MM with the control carbon substrate and containing 20 µg mL^−1^ kanamycin and incubated at 29°C in the dark to select for *C. sphaeroides* transposon mutants. Higher dilutions, 1:10^5^ and 1:10^6^, were plated on MM with the control carbon substrate and without kanamycin to determine conjugation/transposition efficiencies, typically in the range of 1:50,000–1: 100,000.

### Screening of mutants

To discover transposon mutants with a distinct phenotype from wild-type *C. sphaeroides*, as to their ability to use a specific carbon substrate, two different screens were performed. In a primary screen, colonies obtained from the initial conjugation plate were patched in sequence (using sterile wooden toothpicks) on four or five MM-kan agar plates with each plate containing a different carbon substrate, and the plates were incubated aerobically at 29°C in the dark. The last plate in the sequence was used as the control plate containing the same carbon substrate as the initial conjugation plate. For the specific carbon substrates used in the independent rounds of mutagenesis experiments, please see the footnote in Table S1. Scoring of mutants was conducted by visually inspecting the growth of each mutant on one plate and comparing it to the growth of the other 49 mutants on the same plate and across plates with different carbon substrates. Typically, all plates were inspected twice, at 1 week and after more than 2 weeks of incubation. A no-growth phenotype was determined if growth on the control plate was positive, but no colonies (or a few individual colonies on the diagonal streak as a sign of possible suppression) on one or more of the other plates were detected. Compromised growth was assigned as significantly diminished growth throughout the diagonal streak, compared to the growth of other mutants on the same plate, and with the control plate showing robust growth. After mapping the transposon insertion site, a secondary screen was conducted. Mutants of interest were revived from minus 75°C glycerol stock cultures and plated on either MM succinate-kan or MM D/L-lactate-kan, dependent on what was used as a control substrate initially and incubated aerobically at 29°C in the dark. Single colonies were transferred to a 350 μL-microtiter plate well, containing 130 µL MM succinate-kan or MM D/L-lactate-kan. A gas-permeable seal was placed on top of the 96-well microtiter plate, and it was incubated aerobically (shaking) at 29°C in the dark. A sterile metal replicator was used to pick up cells from the liquid medium and to stamp them in sequence on agar trays, each containing minimal medium with a different carbon substrate. A gas-permeable seal was placed on top of the plates, and they were incubated aerobically at 29°C in the dark or anaerobically (using a jar flushed with nitrogen and adding a gas pack [Gas Pak EZ, Becton, Dickinson and Company]) in the light. Carbon substrates included in the screen included acetate, 3-hydroxypropionate, propionate/HCO_3_^–^, L-malate, succinate, and D/L-lactate. Again, scoring for negative or compromised growth was done by visual inspection.

### Identification of transposon insertion sites

Genomic DNA was isolated from *C. sphaeroides* transposon mutant strains and restricted by *Nco*I or *Bam*HI endonuclease. After inactivation of the enzyme by heat, the fragments were circularized using T4 DNA ligase and introduced into *E. coli* strain DH5αλ*pir*. Only circularized fragments containing the transposon, which carries *ori*R6K (and flanking genome sequences), replicate as plasmids. The plasmids were isolated and sequenced using outward-directed transposon-directed primers (13). Insertion of the transposon results in the duplication of an 8–9 nucleotide genomic region at the insertion site. The center of the duplicated sequence was reported as the genomic insertion site.

### Isolation of the marker-less *rsp_3293-3297* deletion strain RsΔABC35KB

The sequences of all primers used in this study are listed in Table 1. The suicide plasmid pKB90, employed for the marker-less deletion of *rsp_3293-3297* (RsΔABC35KB), was constructed by amplifying 142 bp of the 5′ *rsp_3297* coding region plus 1,353 bp directly upstream and 135 bp of the 3′ *rsp_3293* coding region plus 1,083 bp directly downstream of *rsp_3293* and cloning the products in tandem into pK18mobsacB (39). The resulting plasmid pKB90 contains a deletion of 4,286 bp. The remaining open reading frame encodes a 93 amino acid peptide, consisting of a truncated N-terminal RSP_3297 and a truncated C-terminal RSP_3293. *E. coli* S17-1 (40) was transformed with pKB90 and conjugated with *C. sphaeroides* as described above. Single crossover strains of *C. sphaeroides*, that had pKB90 inserted into the genome, were selected by plating the transconjugants on MM-succinate-kan plates. Incubation of a colony of a single crossover strain in the absence of kanamycin allowed for the second crossover to occur, and the cells were plated on MM-succinate-10% sucrose to select for the absence of the plasmid.

**TABLE 1:** List of primers used in this study

Primer	DNA sequence (5′ → 3′)
	Transposon sequencing primers (13)
tnpRL17_1	AACAAGCCAGGGATGTAACG
tnpRL13_2	CAGCAACACCTTCTTCACGA
	*dddC* upstream fragment for deletion
deltadddCup_for1	AAAGGCAAGCTTACGCATCGCGGGACAGG
deltadddCup_rev1	ACGCGGGTACCGTCGATCCAGTGGCTGAG
	*dddC* downstream fragment for deletion
deltadddCdown_for1	GCATCAAGGAGGGTACCGCCTTCAACTTC
deltadddCdown_rev1	TGCGGAGTCATATGTCGATGCCGCCGACGTTGGTC
	*dddC* complementation
dddCexp_NdeIF	CCGAGGGAGATTCATATGGAAGAACTCAG
dddCexp_BamHIR	GAGGTGGATCCGGACCGAAACTCAG
	*rsp_3293-3297* downstream fragment for deletion
deltaABCup_for1	AACTACGGTACCACCTCGCAATTCCCGGTCACG
deltaABCup_rev1^*a*^	TGTCGATCTAGACCGGCTGGACATGGAAC
	*rsp_3293-3297* upstream fragment for deletion
deltaABCdown_for1	ATAGGAATTCTCCATGGCGCCGCCCGAATC
deltaABCdown_rev1	TTAGTAGGTACCGCTGTATTTGCCGCCGAAGTC
	*rsp_3292* upstream fragment for deletion
deltaGmcorup_for1	TCAAAGCTTCGCTGCTTCTGACCTTCACCTACG
deltaGmcorup_rev1	GCGGTTCGAGGTACCACTTGGACAGGCGGTTC
	*rsp_3292* downstream fragment for deletion
deltaGmcordown_for1	TAGCCTCAGGTACCCGGATGCGTCGATCATGC
deltaGmcordown_rev1	TGTCGCCCATATGTCTAGAATTGATGGCAAAGGAG
	*rsp_3292* complementation
dddAexp_NdeIF	GTCGGAGAGCTCATATGGAGGCCGATTAC
dddAexp_HindIIIR	CCCGGGAAAGCTTCATCCTCAAACC
	*rsp_1413* upstream fragment for deletion
delta1413up_for1	CAAGAGGTACCGCCAGAGCAGGTTTCCCTGTTC
delta1413up_rev1	CGTAAGAAGCTTGCAAGGCAACGCACAAATTCC
	*rsp_1412* downstream fragment for deletion
delta1412down_for1	CGTCGGAATTCTCCCGCACATGACCTCATCG
delta1412down_rev1	CATCTTGGTACCGGAGCCGAAGCAGCTCTTCTACATTC
	*rsp_1413/1412* complementation
TRAPcomp_rev6	TACGATCTAGAGATTGTCGAGCAGCCAGACC
TRAPcomp_for4	TTGGAATTCGCTCAGAAGGCGGGCTTG

^*a*^ The *dam* methylation site was later changed by site-directed mutagenesis to AATC. Restriction enzyme recognition sites are underlined.

### Isolation of the marker-less and in-frame *rsp_3292* deletion strain RsΔ*gmor*47KB and complementation

The suicide plasmid pKB60, employed for the marker-less deletion of *rsp_3292*, was constructed by amplifying 69 bp of the 5′ *rsp_3292* coding region plus 1,307 bp directly upstream and 114 bp of the 3′ *rsp_3293* coding region plus 1,305 bp directly downstream of *rsp_3293* and cloning the products in tandem into pK18mobsacB (39). The resulting plasmid pKB60 contains an in-frame deletion of *rsp_3292* of 1,419 bp. The remaining open reading frame encodes a 62-amino acid peptide. Mutant isolation was carried out as described above for RsΔABC35KB. For the complementation of RsΔ*gmor*47KB, *rsp_3292* was amplified from *C. sphaeroides* 2.4.1 genomic DNA, and the 1,611 bp product was cloned into pMA5-1, replacing the *acuI* insert, and placing it under the control of a constitutive *tetA* promoter (5), resulting in plasmid pDO1. *E. coli* SM10 (40) was transformed with pDO1 and conjugated with *C. sphaeroides* as described above, and transconjugants of *C. sphaeroides* were selected using MM succinate containing spectinomycin.

### Isolation of the marker-less and in-frame *dddC*(*rsp_2962*) deletion strain RsΔ*dddC*MA4 and complementation

For the marker-less in-frame deletion of *dddC* (*rsp_2962*), the suicide plasmid pMA10-4 was constructed by amplifying 27 bp of the 5′ *dddC* coding region plus 1,523 bp directly upstream and 27 bp of the 3′ *dddC* coding region plus 1,461 bp directly downstream of *dddC* by PCR and cloning the products (using an internal *Bam*HI site) in tandem into pK18mobsacB (39). The resulting plasmid pMA10-4 contains an in-frame deletion of *dddC* of 1,446 bp. The remaining open reading frame encodes a 19-amino acid peptide. Mutant isolation was carried out as described above for RsΔABC35KB. For the complementation of RsΔ*dddC*MA4, the *dddC* (*rsp_2962*) gene was amplified from *C. sphaeroides* 2.4.1 genomic DNA. The 1,536 bp product was cloned into pMA5-1, replacing the *acuI* insert, and placing it under the control of a constitutive *tetA* promoter (5) resulting in plasmid pMA32. Introduction of the plasmid into *C. sphaeroides* was carried out as described above for the complementation of RsΔ*gmor*47KB.

### Isolation of the marker-less and in-frame *rsp_1413/1412* deletion strain RsΔ*1412_13*SK7 and complementation

For the marker-less in-frame deletion of *rsp_1413/1412*, the suicide plasmid pKB153 was constructed by amplifying 26 bp of the 5′ *rsp_1413* coding region plus 1,188 bp directly upstream and 94 bp of the 3′ *rsp_1412* coding region plus 1,190 bp directly downstream of *rsp_1412* by PCR and cloning the products in tandem into pK18mobsacB (39). The resulting plasmid pKB153 results in a 3,569 bp genomic deletion. Instead of *rsp_1413 and rsp_1412*, a new open reading frame encodes a 41-amino acid peptide, consisting of a 9-amino acid N-terminal sequence of Rsp_1413 and a 29-amino acid C-terminal sequence of Rsp_1412 with three additional amino acids (ValProGlu) in between. Mutant isolation was carried out as described above for RsΔABC35KB. For the complementation of RsΔ*1412_13*SK7, the *rsp_1413* gene plus 244 bp of its upstream region, together with the *rsp_1412* gene, was amplified from *C. sphaeroides* 2.4.1 genomic DNA. The 3,933 bp product was cloned into pBBRsm2MCS5(MC), placing the *rsp_1413/1412* genes under their native promoter, resulting in plasmid pJAA012. Introduction of the plasmid into *C. sphaeroides* was carried out as described above for the complementation of RsΔ*gmor*47KB.

## SUPPLEMENTAL MATERIAL

Supplemental table and figures jb.00306-25-s0001.pdf Table S1 and Figures S1 to S5

ASM does not own the copyrights to Supplemental Material that may be linked to, or accessed through, an article. The authors have granted ASM a non-exclusive, world-wide license to publish the Supplemental Material files. Please contact the corresponding author directly for reuse.
