# Supplementary material for: Probing the core metabolism of Cereibacter sphaeroides by transposon mutagenesis
Source: J Bacteriol. 2025 Oct 30;207(11):e00306-25. doi: 10.1128/jb.00306-25 (PMC12632250; doi:10.1128/jb.00306-25)
Supplement: Supplemental table and figures — Table S1 and Figures S1 to S5 [file jb.00306-25-s0001.pdf]

**Table S1: Transposon insertions at loci with two confirmed hits and consistent plate phenotypes.**

| Strain | Insertion site (CP000143*) | Plate phenotype | Gene *rsp* |
|---|---|---|---|
| Au12-316-4-43 | 13041s | 3-hydroxypropionate-negative (light), | *1434* |
| Sp10-BA36 | 13709s | severely compromised aerobically 3-HP | (*acuI*) |
| | | | |
| ABC21CM97 | 104701s | pale/whitish, phototrophic mutant | *1518* |
| Au14-7pig | 104954s | | (*prrA*) |
| | | | |
| Au12-316-14-141 | 155447s | pale/whitish, phototrophic mutant | *1565* |
| Au14-20PI | 156014s | | (*appA*) |
| Au12-316-17-251 | 156317s | | |
| Sp22EF-177 | 155797s | | |
| | | | |
| ABC21BM9 | 260476s | sporadically showing compromised | *1670* |
| Au12-332-18-247 | 260928s | growth with acetate, 3-hydroxypropionate | *(relA)* |
| Au12-332-1-132 | 261673s | butyrate/$CO_2$; pale and red colonies | |
| | | | |
| Au10-17-85 | 618155o | acetate-, butyrate/$CO_2$-, | *2015* |
| ABC21BM119 | 617376s | 3-hydroxypropionate-negative | (*meaB*) |
| | | | |
| ABC21AR32 | 685100s | D-lactate, D-malate, L-lactate-negative | *2090* |
| Au09MW-28-52 | 685263s | | (*pycA*) |
| Au09TR-23-48 | 685313s | | |
| Au09TR-09-187 | 686065s | | |
| Au09TR-17-143 | 686697s | | |
| Au09TR-34-134 | 686937s | | |
| Sp21CM11 | 687161s | | |
| Feb21AR170 | 687513s | | |
| | | | |
| Au12-316-34-26 | 796440s | propionate/$CO_2$- butyrate/$CO_2$-negative, | *2189* |
| Sp11-29-24 | 796513s | 3-hydroxypropionate-negative | (*pccB*) |
| Au12-316-14-76 | 796603s | acetate (slightly) compromised | |
| Sp11-36-126 | 796743s | | |
| Sp10-35-256 | 800097s | propionate/$CO_2$- butyrate/$CO_2$-negative, | *2191* |
| Au12-332-5-165 | 800390s | 3-hydroxypropionate-compromised | (*pccA*) |
| Au11-9-39 | 800886s | acetate (slightly) compromised | |
| Au12-332-3-8 | 801237s | | |
| Ap19ID168 | 801255s | | |
| Au14-17-132 | 802254s | acetate-, 3-hydroxypropionate-, butyrate/ | *2192* |
| Au09TR10-48 | 802869s | $CO_2$-, propionate/$CO_2$-negative | (*mcm*) |
| Au12-316-33-37 | 803827s | slightly slower growth in general | |
| Au12-316-8-64 | 804085s | | |
| Au10MW30-25 | 804122s | | |
| | | | |
| ABC21CM46 | 1123027s | L/D-lactate-, L-malate-negative | *4049* (*acoA*) |
| ABC21BM169 | 1125039s | L/D-lactate-, L-malate-negative | *4047* (*acoB*) |
| | | | |

| | | | |
|---|---|---|---|
| Au11-7-114 | 1644465s | 3-hydroxypropionate-negative | *2962* |
| Sp19MD38 | 1645323s | | (*dddC*) |
| Au12-316-6-153 | 1645477s | | |
| Au12-316-5-65 | 1645875s | | |
| Sp10MA1-14 | 1645923s | | |
| Au12-332-23-43 | 1645923s | | |
| Au09TR28-20 | 1645949s | | |
| Feb21AR122 | 1645069s | | |
| | | | |
| Au18KH88pig | 1984256s | phototrophic mutant, orange | *0260* |
| Au10MW18-28 | 1984587s | | (*bchZ*) |
| Au10TR15-63 | 1986178s | phototrophic mutant, orange/whitish | *0261 (bchY)* |
| Au11-5-198 | 1989972s | phototrophic mutant, whitish | *0265 (crtE)* |
| | | | |
| Sp10-32-10 | 1990719s | light green, phototrophic competent, | *0266* |
| Sp11-28-26 | 1191747s | aerobically compromised | (*crtD*) |
| Sp10-MA6-1 | 1991961s | | |
| Sp19MD32 | 1992495s | green, phototrophic competent, aerobically compromised | *0267 (crtC)* |
| | | | |
| Sp11-KS11 | 1995123s | gray, phototrophic competent | *0270* |
| Au11-16-132 | 1995152s | | (*crtI*) |
| | | | |
| Au09-TR31pig | 1997199s | phototrophic mutant, pale to orange | *0272* |
| Au12-316-5PG | 1997327s | | (*crtA*) |
| | | | |
| Au14-21pig | 2002843s | phototrophic mutant, orange/yellow | *0277* (*bchP*) |
| | | | |
| Au10MW5-01 | 2014010s | phototrophic mutant, pale to orange | *0287* |
| Sp10-28-6 | 2014491s | | (*bchH*) |
| Au14-22-25 | 2015593o | | |
| | | | |
| Sp11-18-162 | 2130076s | L-glutamate-compromised | *0398* |
| Sp11-9-215 | 2130325s | | (*gdh*) |
| | | | |
| Au09T2-101 | 2392021s | acetate-compromised | *0653* |
| Sp23NJB6-33 | 2391779s | | |
| | | | |
| Au14-12-6-84 | 2489933o | acetate-negative | *0745* |
| Au18OM70 | 2489679s | | (*phaA*) |
| Sp23NJB2-23 | 2490005s | | |
| Au12-316-23-247 | 2491174s | acetate-negative | *0747* |
| Au12-316-26-2 | 2491369s | | (*phaB*) |
| | | | |
| Au14-22-162 | 2659915s | L-malate-, succinate-negative | *0910* |
| Sp10-27-29 | 2660431s | | (*dctP*) |
| | | | |
| Au12-332-22-1 | 2661665o | L-malate-, succinate-negative | *0912* |
| Sp10-35-204 | 2662641s | | (*dctM*) |

| | | | |
|---|---|---|---|
| Sp18KK51 | 2734023s | acetate-negative, butyrate/$CO_2$-negative | *0973* |
| Sp10BA206 | 2734509s | 3-hydroxypropionate-negative (aerobically) | (*mch*) |
| | | | |
| **Strain** | **Insertion site (CP000144*)** | **Phenotype** | **Gene (*rsp_*)** |
| Sp11MA16-39 | 354953o | 3-hydroxypropionate compromised/ | *3295* |
| Sp11MA26-40 | 355315s | negative | *3296* |
| Sp11JAA14-4 | 355650o | | |
| | | | |
| Au11-38-158 | 927771s | L-glutamate-negative | *1412* |
| Sp11-18-60 | 928115s | | |
| | | | |

*Sequence download: 12-FEB-2007. The designation "s" or "o" after the insertion site, indicates that the gene encoding aminoglycoside phosphotransferase ($kan^R$) is transcribed from the same (s) or complementary (o) coding strand in respect to the interrupted gene. Compromised growth is defined as impaired growth compared to the growth for other mutants on the same plate.

#The following carbon substrates were used to screen transposon mutants (primary screen) and plates were incubated aerobically in the dark:

**Au09**: D/L-lactate, acetate, acrylate, and succinate (control)
**Sp10**: butyrate/$HCO_3^-$, 3-hydroxypropionate, L-malate, propionate/$HCO_3^-$, and D/L-lactate (control)
**Au10**: butyrate/$HCO_3^-$, propionate/$HCO_3^-$, acetate, L-lactate, and succinate (control)
**Sp11**: butyrate/$HCO_3^-$, L-glutamate, L-isoleucine/$HCO_3^-$, succinate, and D/L-lactate (control)
**Au11**: L-glutamate, acetate, 3-hydroxypropionate, L-malate, and D/L-lactate (control)
**Au12**: acetate, L-alanine, 3-hydroxypropionate, L-glutamate, L-malate, and D/L-lactate (control)
**Au14**: L-glutamate, acetate, propionate/$HCO_3^-$, L-malate, succinate, and D/L-lactate (control)
**Sp18**: D-lactate, acetate, D-malate, L-lactate, L-malate (control)
**Au18**: D-malate, acetate, L-lactate, D-lactate, and L-malate (control)
**Sp19**: acetate, 3-hydroxypropionate, D-malate, L-malate, and D/L-lactate (control)
**Feb21**: L-malate, D-lactate, L-lactate, 3-hydroxypropionate, acetate/$HCO_3^-$ (control)
**Sp21**: L-lactate, L-malate, L-malate/$Na_2S$, D-lactate, acetate/$HCO_3^-$ (control)
**ABC21**: L-lactate, L-malate, L-malate/$Na_2S$ or 3-hydroxypropionate, D-lactate, acetate/$HCO_3^-$ (control)
**Sp22**: acetate, L-lactate, D-lactate, succinate, and L-malate (control)
**Sp23**: methanol/$HCO_3^-$, acetate, L-glutamate, D-lactate, and L-malate (control)

Most mutants were subjected to a secondary screen and carbon substrates included were acetate, L-malate, propionate/$HCO_3^-$, 3-hydroxypropionate, succinate, and D/L-lactate and plates were incubated aerobically in the dark and another set of plates was incubated anaerobically in the light.

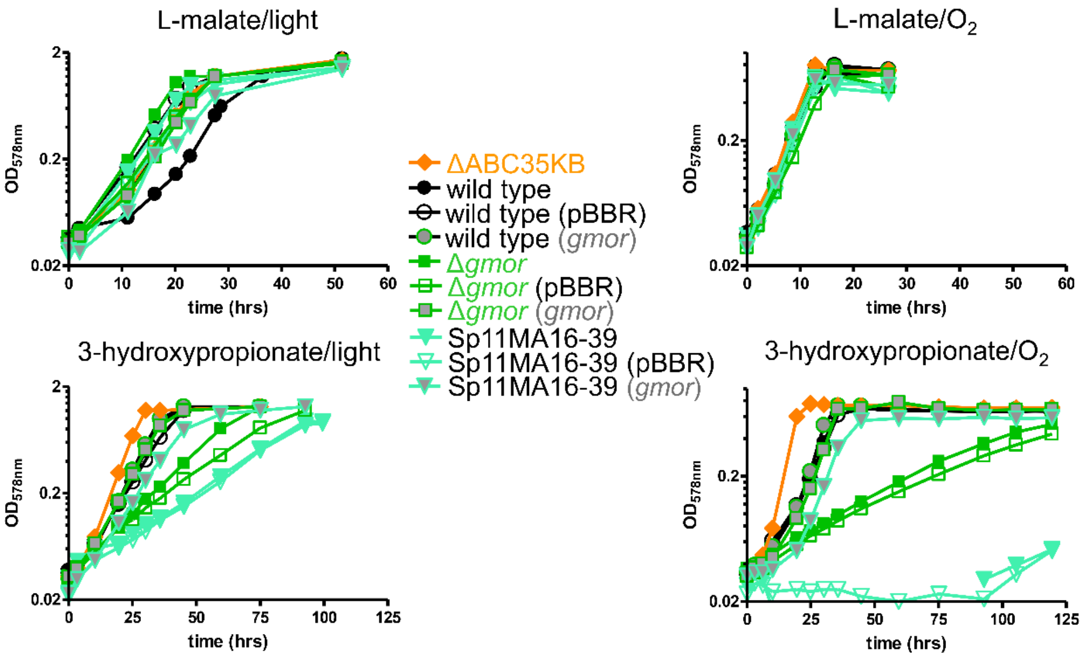


| | L-malate/light | L-malate/$O_2$ | 3-hydroxy-propionate/light | 3-hydroxy-propionate/$O_2$ |
|---|---|---|---|---|
| ΔABC35KB | 3.6 (2) | 2.6 (2) | 5.0 ± 0.2 (3) | 3.7 ± 0.3 (3) |
| wild type | 3.7 ± 0.2 (3) | 2.6 (2) | 6.0 ± 0.5 (6) | 6.4 ± 0.5 (5) |
| wild type (pBBR) | 4.6 ± 0.5 (3) | 3.1 ± 0.2 (3) | 6.8 ± 1.0 (5) | 6.8 ± 0.4 (5) |
| wild type (*gmor*) | 4.3 (2) | 2.7 (2) | 5.5 ± 1.0 (4) | 5.2 ± 0.5 (3) |
| Δ*gmor* | 3.8 ± 0.2 (3) | 2.6 (2) | 12 ± 1.0 (6) | 30 ± 5.2 (5) |
| Δ*gmor* (pBBR) | 4.9 (2) | 3.3 (2) | 14 ± 2.8 (4) | 26 ± 1.8 (4) |
| Δ*gmor* (*gmor*) | 4.0 (2) | 2.8 (2) | 5.7 ± 0.4 (5) | 5.8 ± 0.4 (5) |
| Sp11MA16-39 | 4.1 (1) | 2.6 (1) | 18 (1) | 34 (1) clumps |
| Sp11MA16-39 (pBBR) | 4.5 (2) | 2.8 (1) | 20 (2) | 27 (1) clumps |
| Sp11MA16-39 (*gmor*) | 4.6 (2) | 2.8 (2) | 9.7 (2) | 5.5 (2) |

**Fig. S1** Growth of the wild-type, the Δ*gmor*47KB and the transposon mutant Sp11MA16-39 strains, carrying no plasmid, an empty vector control (pBBR) or the intact *gmor* gene on a pBBR-derived plasmid (*gmor*), either anaerobically in the light (photoheterotrophically, left graphs) or aerobically in the dark (respiratory growth, right graphs) with either L-malate (top) or 3-hydroxypropionate (bottom) as the carbon source. For aerobic respiratory growth of Sp11MA16-39 with 3-hydroxypropionate clumping of the cells was observed. Although Sp11MA16-39 carries a transposon in *rsp_3295*, encoding a subunit of the ABC transporter, introduction of the downstream *gmor* (*rsp_3292*) gene, encoding a possible 3-hydroxy-propionate dehydrogenase, restores growth with 3-hydroxypropionate. Average doubling times from several growth experiments are provided in the table (number of replica are shown in parentheses and standard derivations were calculated in the case of three or more replica).

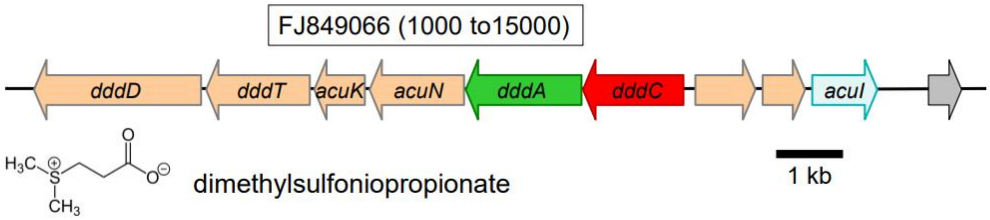


**Fig. S2** Genomic region of *Halomonas* sp. HTNK1 containing genes required for the metabolism of dimethylsulfoniopropionate (DMSP) according to Todd *et al.* 2010. DddD is proposed to cleave DMSP into dimethyl sulfide and 3-hydroxypropionate. The amino acid sequence of DddA from *Halomonas* sp. is 61 % identical to Gmor of *C. sphaeroides* and the two DddC proteins from both organisms are 53 % identical, over the full lengths of the proteins. The amino acid sequences of the gene product of *acuI* from *Halomonas* sp. is 61 % identical to that of acrylyl-CoA reductase from *C. sphaeroides*, an enzyme involved in the so-called reductive route of 3-hydroxypropionate metabolism (Schneider *et al*. 2012, Asao & Alber 2013).

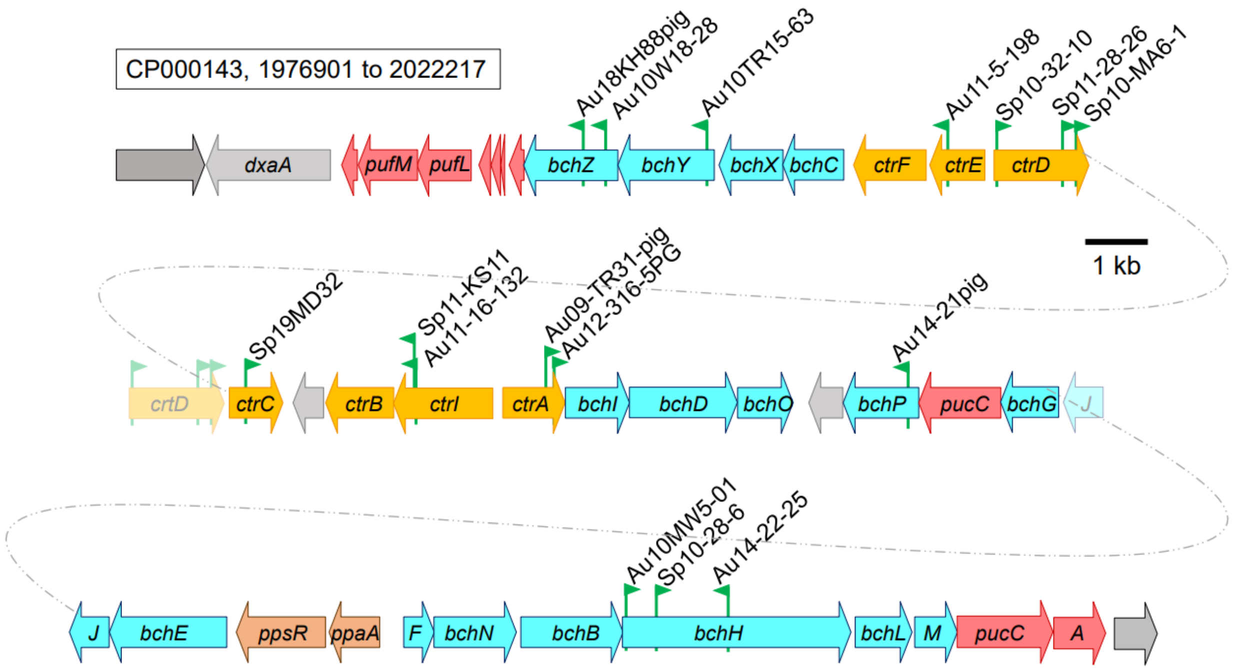


**Fig. S3** Genomic region of *C. sphaeroides* with genes encoding enzymes for the synthesis of carotenoids (*crt* genes indicated in orange), bacteriochlorophyll (*bch* genes indicated in blue), regulatory proteins (in brown) or structural proteins of the reaction center or of the light harvesting complexes (in red). Flags mark the insertion site for each mutant and the arrows indicate the transcriptional direction of the kanamycin resistance marker gene that is part of the transposon. All mutants were unable to grow in the light, except those with a transposon insertion in *crtI, crtC,* or *crtD*.

**Pigmentation mutants.** The ability to use light as a source of energy (phototrophy) is in part due to the presence of the so-called “photosynthesis gene cluster” of *C. sphaeroides*, that encodes enzymes for the biosynthesis of bacteriochlorophyll a and carotenoids, as well as structural components of a reaction center and two light harvesting complexes (**Fig. S3**). Several mutants with a pigmentation on aerobic plates distinct from the wild type were isolated and the insertions mapped to this photosynthesis gene cluster. The insertion of a transposon in any of the *bch* genes, encoding enzymes in the biosynthesis pathway for bacteriochlorophyll, resulted in the inability to use light as a source of energy, as expected. The two *crtD* mutants displayed a light green phenotype consistent with the blockage of the biosynthesis pathway of spheroidene at the level of hydroneurosporene (but additional derivatives are formed, Chi *et al* 2015, Coomber *et al.* 1990); interestingly, both *crtD*-mutants were competent for growth in the light, but growth was compromised aerobically. Chi *et al* (2015) had observed that deletion of *crtD* resulted in what they described as an unstable phenotype. Aerobically compromised but phototrophic competent growth was also observed for the green *crtC* mutant (expected to accumulate neurosporene). The inability of the *crtA* mutants to grow phototrophically may be due to a polar effect on the expression of the downstream *bch* genes or to the inability to form spheroidenone in the presence of some oxygen remaining in the culture.

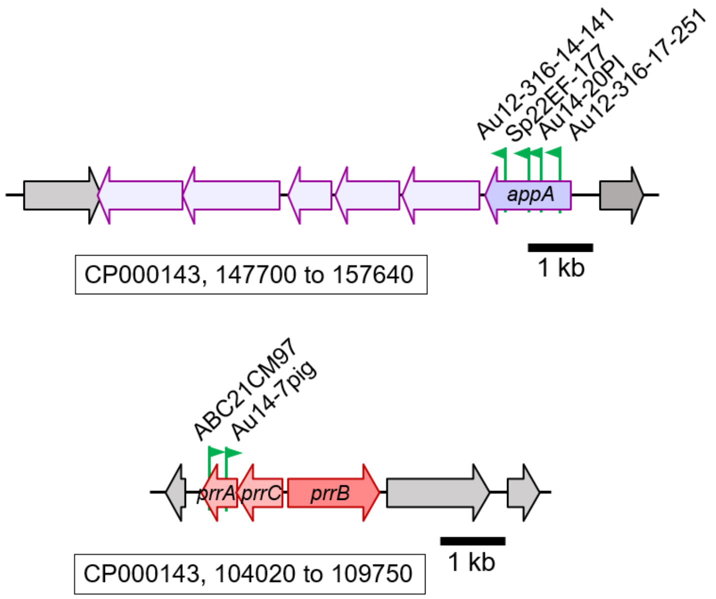


**Fig. S4** Genomic regions of *C. sphaeroides* with genes encoding regulatory proteins required for up-regulation of genes shown in **Fig. S3**, essential for phototrophic growth. Flags mark the insertion site for each mutant and the arrows indicate the transcriptional direction of the kanamycin resistance marker gene that is part of the transposon. All mutants formed white colonies on plates and were unable to grow in the light. The *rsp_1565* gene encodes AppA (accession number ABA77661), an antirepressor of PpsR, which in turn is a repressor of genes involved in carotenoid and bacteriochlorophyll biosynthesis. The *rsp_1518* gene encodes PrrA (named RegA for *Rhodobacter capsulatus*), a DNA-binding response regulator of a two-component system up-regulating several genes during anaerobic growth including those required for phototrophy.

Two phototrophic-negative transposon mutants were isolated, due to their lack of pigmentation, and the insertions were mapped to the gene *rsp_1565* encoding AppA (**Table S1, Fig. S4**). It has been previously shown that the absence of AppA in the cell leads to constitutive repression of the photosynthesis gene cluster" by PpsR and, therefore, to a phototrophic-negative phenotype, whereas overproduction of AppA can partially overcome down-regulation of genes required for phototrophic growth in a *prrA* deletion strain (Gomelsky *et al.* 1995). PrrB-PrrA of *C. sphaeroides* is a histidine kinase-response regulator two component regulatory system and is called RegB-RegA in the case of *Rhodobacter capsulatus* (Eraso & Kaplan, 1994; Sganga & Bauer, 1992). These regulatory systems control the expression of genes required to switch to low-oxygen tension growth, among them, genes of the photosynthesis gene cluster (Elsen *et al*. 2004). It is, therefore, not surprising that the absence of PrrA results in a whitish pigmentation and a phototrophic-negative phenotype (**Table S1**).

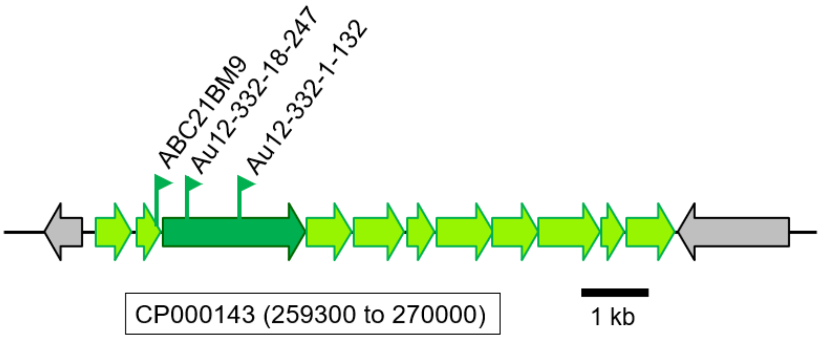


**Fig. S5** Insertion sites for *C. sphaeroides* transposon mutants isolated as sporadically acetate-, and 3-hydroxypropionate-compromised. When streaked for isolation on plates containing these substrates, some individual colonies appeared much paler than most of the normally red colonies present. Flags mark the insertion site for each mutant and the arrows indicate the transcriptional direction of the kanamycin resistance marker gene that is part of the transposon. The *rsp_1670* highlighted encodes a RelA/SpoT homolog (accession number ABA77818).

**The stringent response.** Three transposon mutants were isolated that had a consistent phenotype in occasionally, but not always, showing diminished growth with acetate, 3-hydroxypropionate and butyrate/$HCO_3^-$. When streaked for isolation on plates containing these substrates, some individual colonies appeared much paler than most of the normally red colonies present. The transposon insertion sites were mapped to the *rsp_1670* gene (or just upstream) (**Table S1, Fig. S5**), annotated to encode a RelA-SpoT-homolog. The Rsp_1670 protein (accession number ABA77818) is expected to catalyze the addition of pyrophosphate to the 3'-hydroxyl-group of GTP or GDP to form (p)ppGpp and in addition it contains a protein domain responsible for (p)ppGpp hydrolysis. The loss in maintenance of optimal levels of (p)ppGpp or so-called alarmones, results in the inability to adapt to imbalanced growth caused by rapidly changing conditions. The sporadically changing phenotype of the three transposon mutants may be explained by individual cells in the population failing to maintain optimal (p)ppGpp levels and, therefore, lack balanced growth. Rsp_1670 is identical to the enzyme studied from *Rhodobacter capsulatus*, with an ACT domain that binds branched amino acids to control the (p)ppGpp hydrolase activity (Fang & Bauer 2018); however, additional C-terminal regulatory domains are likely to respond to other cues (Irving *et al.* 2021).

**References (Supplementary Materials)**

Asao M., and B. E. Alber (2013). Acrylyl-Coenzyme A reductase, an enzyme involved in the assimilation of 3-hydroxypropionate by *Rhodobacter sphaeroides. J. Bacteriol.* **195:**4716-4725.

Chi S. C., Mothersole D. J., Dilbeck P., Niedzwiedzki D. M., Zhang H., Qian P., Vasilev C., Gratson K. J., Jackson P. J., Martin E. C., Li Y., Holten D., and C. N. Hunter (2015). Assembly of functional photosystem complexes in *Rhodobacter sphaeroides* incorporating carotenoids from the spirilloxanthin pathway. *Biochim. Biophys. Acta* **1847:**189-201.

Coomber S. A., Chaudhri M., Connor A., Britton G., and C. N. Hunter (1990). Localized transposon Tn5 mutagenesis of the photosynthetic gene cluster of *Rhodobacter sphaeroides*. *Mol. Microbiol*.**4:**977–989.

Elsen S., Swem L. R., Swem D. L., and C. E. Bauer (2004). RegB/RegA, a highly conserved redox-responding global two-component regulatory system. *Microbiol. Mol. Biol. Rev*., **68**:263-279.

Eraso J. M., and S. Kaplan. (1994). PrrA, a putative response regulator involved in oxygen regulation of photosynthesis gene expression in *Rhodobacter sphaeroides*. *J. Bacteriol*. **176:**32–43

Fang M., and C. E. Bauer (2018). Regulation of stringent factor by branched-chain amino acids. *Proc. Natl. Acad. Sci. USA* **115:**6446-6451.

Gomelsky M. and S. Kaplan (1995). *appA*, a novel gene encoding a *trans*-acting factor involved in the regulation of photosynthesis gene expression in *Rhodobacter sphaeroides* 2.4.1. *J. Bacteriol.* **172:**4609-4618.

Irving S. E., Choudhury N. R., and R. M. Corrigan (2021). The stringent response and physiological roles of (pp)pGpp in bacteria. *Nat. Rev. Microbiol.* **19:**256-271.

Schneider K., Asao M., Carter M. S., and B. E. Alber (2012). *Rhodobacter sphaeroides* uses a reductive route via propionyl coenzyme A to assimilate 3-hydroxypropionate. *J. Bacteriol.* **194:**225-232.

Sganga, M. W., and C. E. Bauer. (1992). Regulatory factors controlling photosynthetic reaction center and light-harvesting gene expression in *Rhodobacter capsulatus*. *Cell* **68:**945–954.

Todd J. D., Curson A. R. J., Nikolaidou-Katsaraidou N., Brearley C. A., Watmough N. J., Cha, Y., Page P. C. B., Sun L., and A. W. B. Johnston (2010). Molecular dissection of bacterial acrylate catabolism – unexpected links with dimethylsulfoniopropionate catabolism and dimethyl sulfide production. *Environ. Microbiol.* **12:**327-343.
